# A biophysical account of multiplication by a single neuron

**DOI:** 10.1038/s41586-022-04428-3

**Published:** 2022-02-23

**Authors:** Lukas N. Groschner, Jonatan G. Malis, Birte Zuidinga, Alexander Borst

**Affiliations:** Max Planck Institute of Neurobiology, Martinsried, Germany

**Keywords:** Neural circuits, Motion detection, Biophysical models

## Abstract

Nonlinear, multiplication-like operations carried out by individual nerve cells greatly enhance the computational power of a neural system^1–3^, but our understanding of their biophysical implementation is scant. Here we pursue this problem in the *Drosophila melanogaster* ON motion vision circuit^4,5^, in which we record the membrane potentials of direction-selective T4 neurons and of their columnar input elements^6,7^ in response to visual and pharmacological stimuli in vivo. Our electrophysiological measurements and conductance-based simulations provide evidence for a passive supralinear interaction between two distinct types of synapse on T4 dendrites. We show that this multiplication-like nonlinearity arises from the coincidence of cholinergic excitation and release from glutamatergic inhibition. The latter depends on the expression of the glutamate-gated chloride channel GluClα^8,9^ in T4 neurons, which sharpens the directional tuning of the cells and shapes the optomotor behaviour of the animals. Interacting pairs of shunting inhibitory and excitatory synapses have long been postulated as an analogue approximation of a multiplication, which is integral to theories of motion detection^10,11^, sound localization^12^ and sensorimotor control^13^.

## Abstract (web summary)

Release from shunting inhibition and coincident excitation implement a multiplication-like synaptic interaction in motion-sensing neurons of *Drosophila melanogaster*.

## Main

**Fig. 1:**
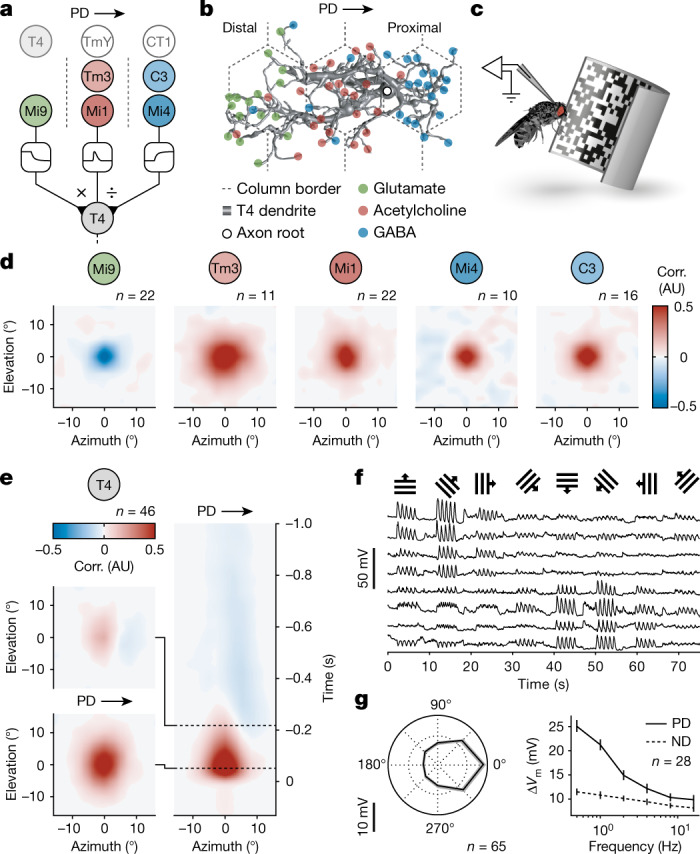
Receptive fields of direction-selective T4 neurons and their presynaptic partners. **a**, The circuit architecture for visual ON motion detection involving a multiplicative interaction (×) between synapses of glutamatergic Mi9 and synapses of cholinergic Mi1/Tm3 neurons and a divisive interaction (÷) between synapses of Mi1/Tm3 and synapses of GABAergic C3/Mi4 neurons. Non-columnar inputs from T4, TmY15 and CT1 neurons are shaded. The dashed lines show the column borders. **b**, A T4 dendrite with subcellular segregation of glutamatergic (green), cholinergic (red) and GABAergic synapses (blue). Data from ref. ^7^. **c**, Targeted patch-clamp recording in vivo during visual stimulation. **d**, Average spatial receptive fields of input neuron classes obtained by reverse correlation (corr.) of membrane potentials and white-noise stimuli. AU, arbitrary units. **e**, The average spatial receptive fields of T4 neurons (left) representing cross-sections of the spatiotemporal receptive field (right) at two time points (dashed lines). **f**, Exemplary membrane potential recordings of T4 neurons in response to visual stimulation with square-wave gratings moving in the directions indicated on top. **g**, Directional (left) and frequency tuning (right) of T4 neurons based on the change in membrane potential (∆*V*_m_) in response to visual stimulation with square-wave gratings. Data are mean ± s.e.m. *n* values indicate the number of cells. Source data.

Motion vision in insects represents a textbook example^14^ of nonlinear signal processing by a single neuron. Each photoreceptor of the compound eye captures changes in light intensity, but it is blind to the direction of motion. To compute visual motion, the signals of at least two neighbouring photoreceptors must be processed nonlinearly by a downstream local motion detector (Fig. 1a). In the Hassenstein–Reichardt model^10^, multiplication ensures detector output only if the two signals coincide. The coincidence results from asymmetric temporal filtering of the input signals and the sequence of photoreceptor activation, one after the other, as it unfolds during visual motion in the detector’s preferred direction (PD). The Barlow–Levick model of motion vision, which was first proposed for the rabbit retina^15^, uses a divisive nonlinearity to cancel responses to motion in the detector’s null direction (ND).

The visual system of *Drosophila* is compatible with both models (Fig. 1a). T4 neurons, which are functionally equivalent to the nonlinear stages of both models, respond selectively to luminance increments moving in one out of four cardinal directions^5^. Their direction selectivity arises in the second optic neuropil^5,16,17^, where spatial information is preserved in a retinotopic columnar organization^18^. Each T4 dendrite innervates approximately seven columns—at least three in a row along the neuron’s PD^6^ (Fig. 1b)—and, therefore, samples from multiple adjacent points in visual space. Recent studies^6,7^ identified most—if not all—columnar medulla intrinsic (Mi), transmedullary (Tm) and centrifugal (C) neurons that form synapses at distinct locations along a T4 neuron’s dendrite: glutamatergic Mi9 neurons at the distal branches (where stimuli moving in the T4 cell’s PD first affect its membrane potential), cholinergic Tm3 and Mi1 neurons at the centre, and GABAergic Mi4 and C3 neurons at the proximal segment (Fig. 1b). The emerging three-legged circuit motif involves a divisive interaction between cholinergic and GABAergic synapses and a multiplicative interaction between glutamatergic and cholinergic synapses^17,19–22^ (Fig. 1a, b). However, crucial assumptions concerning the multiplicative term of this model^21^ remain untested: (1) the multiplication-like synaptic interaction involves disinhibition; (2) the supralinearity arises from the T4 cells’ passive membrane properties; and (3) it sharpens the directional tuning of the neurons and the optomotor acuity of the animal.

**Extended Data Fig. 1:**
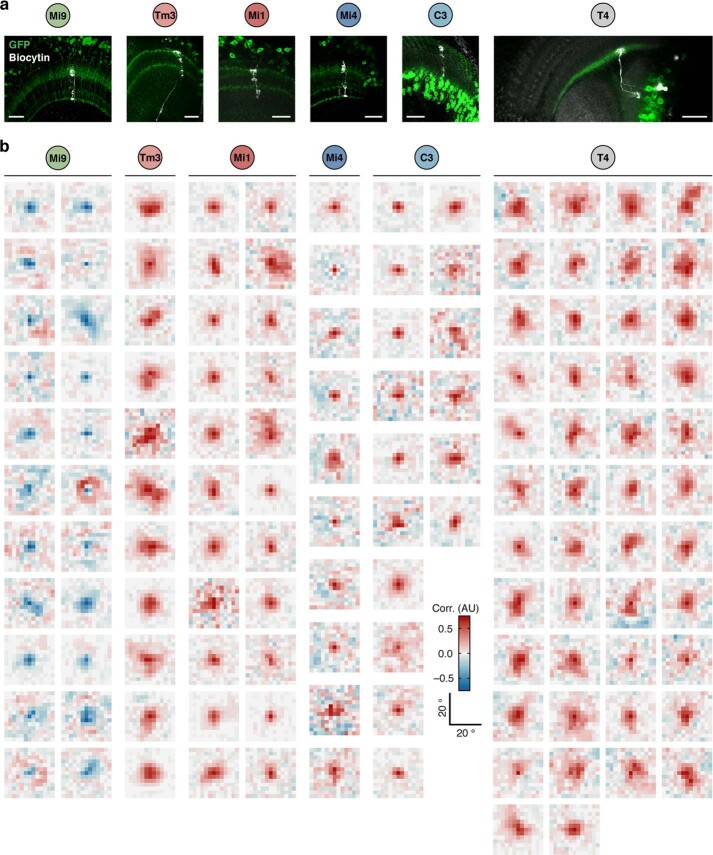
Neuronal morphologies and receptive fields of the ON motion detection circuit. **a**, Maximum intensity projections of confocal stacks with GFP expression in the respective neuronal population (green) and single biocytin-filled neurons (white) recovered after patch-clamp recordings. Scale bars, 20 μm. Micrographs are representative of independent experiments in different flies (Mi9: *n* = 5, Tm3: *n* = 3, Mi1: *n* = 3, Mi4: *n* = 4, C3: *n* = 3, T4: *n* = 7). **b**, Individual spatial receptive fields of T4 and their columnar input neurons obtained by reverse correlation (corr.) of membrane potentials and white noise stimuli. AU, arbitrary units. Filtered averages are shown in Fig. 1d, e. Source data

**Extended Data Fig. 2:**
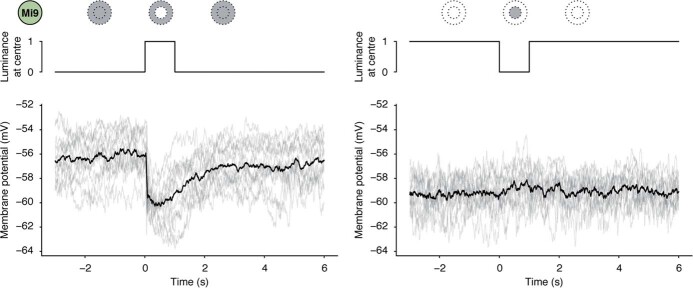
Mi9 neurons hyperpolarize in response to luminance increments. Membrane potential responses of the same Mi9 neurons to increments (left) and decrements in luminance (right) presented in a 5° circle at the centre of the neurons’ receptive fields on a dark or bright background, respectively. Traces on top are normalized light intensities at the respective receptive field centre. The light lines represent technical replicates; the dark lines represent the mean; *n* = 14 technical replicates/2 cells/2 flies. Note the difference in membrane potential depending on the baseline luminance. Source data

The first assumption, that multiplication requires release from inhibition, hinges on the conditions that the signals carried by glutamatergic Mi9 neurons are of opposite polarity to those of the other input elements and that glutamate controls the input resistance of T4 neurons through shunting inhibition^23^. Direct measurements of input resistance and membrane voltage are possible only through patch-clamp experiments, which we conducted in vivo in tethered flies, guided by cell-type-specific expression of green fluorescent protein (GFP; Extended Data Fig. 1a). We recorded the membrane potentials of T4 cells and of their presynaptic partners while projecting a 60 Hz spatiotemporal binary white-noise stimulus with a pixel size of 2.8° onto the fly’s eye. To characterize the receptive fields of the neurons, we cross-correlated the luminance of each pixel with the recorded voltage (Fig. 1c–e and Extended Data Fig. 1b). We found that the membrane potentials of Tm3, Mi1, Mi4 and C3 neurons were positively correlated with luminance, whereas those of Mi9 neurons were anticorrelated (Fig. 1d). The negative correlation was due to a rapid hyperpolarization following increments in luminance, as opposed to a possible depolarization in response to luminance decrements (Extended Data Fig. 2). Thus, the Mi9 neuron maintains a degree of continuous activity in darkness that ceases abruptly when the centre of its receptive field is stimulated by light. Yet, while the delayed inhibition mediated by GABAergic inputs^24^ was clearly discernible in the spatiotemporal receptive fields of direction-selective T4 neurons (Fig. 1e–g), the contribution of Mi9 neurons was not immediately apparent.

**Fig. 2:**
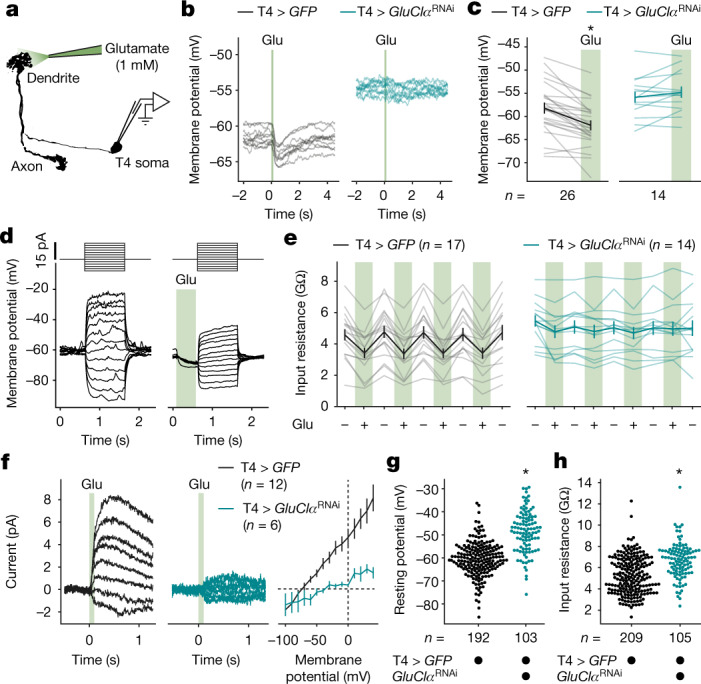
Glutamate controls T4 neuron excitability through GluClα. **a**, Glutamate application during whole-cell recording. **b**, Membrane potential traces of exemplary T4 neurons in response to 100 ms glutamate pulses (Glu) in flies expressing *GFP* (black; T4 > *GFP*, full genotypes are provided in the Methods) or *GFP* + *GluClα*^RNAi^ (teal; T4 > *GluClα*^RNAi^) under T4-cell-specific GAL4 control. Ten technical replicates per genotype are shown. **c**, The average membrane potentials of T4 neurons expressing *GFP* (black) or *GFP *+ *GluClα*^RNAi^ (teal) before and after glutamate application (green). A significant effect of glutamate, determined using a two-tailed paired Student’s *t*-test, is indicated; *P = 2.1 × 10^−6^. The light lines represent individual cells. The dark lines represent the mean ± s.e.m. **d**, Voltage responses of one exemplary T4 neuron to current steps (top) without (left) and with (right) prior glutamate application. **e**, Input resistances of T4 neurons expressing *GFP* (black) or *GFP* + *GluClα*^RNAi^ (teal) during (+) and in between (–) repeated glutamate applications. The light lines represent individual cells. The dark lines represent the mean ± s.e.m. Two-way repeated-measures analysis of variance (ANOVA) detected a significant effect of glutamate (*P* = 3.5 × 10^–12^) and a significant glutamate × genotype interaction (*P* = 1.6 × 10^–11^). **f**, Average whole-cell currents in response to 100 ms glutamate pulses at different voltages (left and middle) and current–voltage relationships (right) of T4 neurons expressing *GFP* (black) or *GFP* + *GluClα*^RNAi^ (teal). Data are mean ± s.e.m. **g**, **h**, Resting membrane potentials (**g**) and input resistances (**h**) of T4 neurons expressing *GFP* (black) or *GFP* + *GluClα*^RNAi^ (teal) measured under dark conditions. Significant differences between genotypes, determined using two-tailed Mann–Whitney *U*-tests, are indicated; *P = 3.4 × 10^−23^ (**g**), *P = 4.8 × 10^–11^(**h**). *n* values indicate the number of cells. Source data.

**Extended Data Fig. 3:**
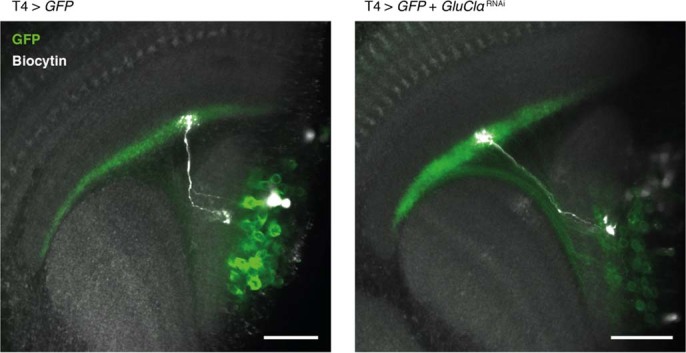
Morphology of wild-type and GluClα-deficient T4 neurons. Maximum intensity projections of representative confocal stacks of T4 neurons expressing *GFP* (left) or *GFP* and *GluClα*^RNAi^ (right), each containing an individual biocytin-filled neuron (white) recovered after patch-clamp recording. The soma of the *GluClα*^RNAi^-expressing neuron was lost during pipette removal. Scale bars, 20 μm. Micrographs are representative of independent experiments in different flies (T4 > *GFP*: *n* = 7 and T4 > *GFP* + *GluClα*^RNAi^: *n* = 3). At the light microscopic level, no obvious genotype-specific morphological differences were detectable.

To test the effect of glutamate—and, indirectly, that of Mi9—on T4 neurons, we applied the neurotransmitter directly to T4 dendrites (Fig. 2a). Pneumatic ejection of glutamate transiently hyperpolarized T4 cells by 3.72 ± 0.61 mV (mean ± s.e.m.; Fig. 2b, c). The mild hyperpolarization was paralleled by a 25.27% decrease in input resistance, which was fully reversible. Repeated applications of glutamate enabled us to toggle T4 cells between states of high and low resistance (Fig. 2d, e). Targeted RNA interference (RNAi) with transcripts of *GluClα*^8^, the most highly expressed glutamate receptor gene in T4 neurons^25–28^, blocked glutamate-gated whole-cell currents (Fig. 2f) and abolished the effects of glutamate on membrane potential and input resistance (Fig. 2b, c, e), while leaving the morphology of T4 cells intact (Extended Data Fig. 3). Importantly, post-transcriptional silencing of *GluClα* caused an average 11.94 mV depolarization of the resting membrane potential (Fig. 2g) and an increase in input resistance from 5.28 ± 0.12 to 6.70 ± 0.16 GΩ (mean ± s.e.m.; Fig. 2h), measured under dark conditions. This speaks for a persistent release of glutamate in the dark that keeps GluClα channels open and clamps the membrane potential of T4 neurons close to the equilibrium potential of chloride—a GluClα-mediated short circuit that curtails any excitation, unless glutamatergic Mi9 neurons are switched off first.

**Fig. 3:**
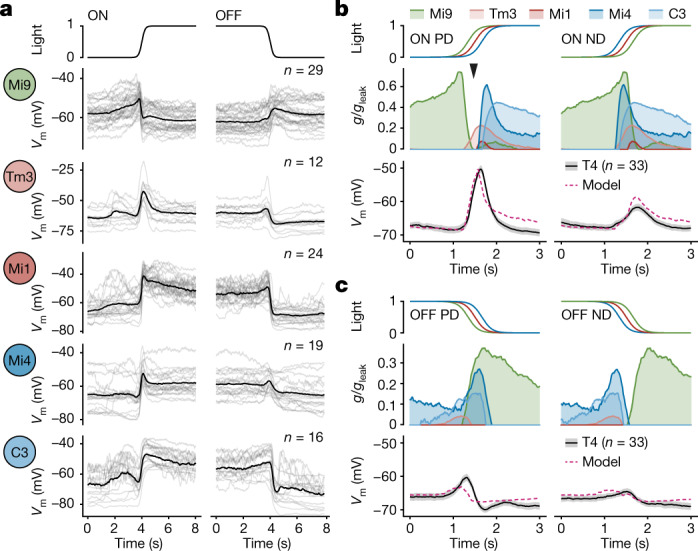
Conductance-based T4 neuron model. **a**, Aligned membrane voltage (*V*_m_) responses of columnar T4 input neurons to ON and OFF edges moving at 30° s^−1^. Time course of normalized light intensity at the receptive field centre is shown at the top. The light lines represent individual cells. The dark lines represent the mean. **b**, **c**, Conductance-based biophysical simulations of the membrane voltage (*V*_m_) of a T4 neuron in response to ON (**b**) and OFF (**c**) edge motion. Input signals were time-shifted, as evident from light intensities at receptive field centres (top), to simulate visual motion in the T4 neuron’s PD and ND, respectively. The voltage signals of presynaptic neurons were converted into normalized postsynaptic conductances (*g*/*g*_leak_, centre) using a threshold and gain obtained by fitting the model (dashed pink) to measured T4 voltage responses (solid black, bottom). Conductance values are mean and area under curve. Voltage values are mean ± s.e.m. The arrowhead in **b** marks the window of opportunity when a minimum of shunting inhibition (green/blue) coincides with excitation (red). *n* values indicate the number of cells. Source data.

**Extended Data Fig. 4:**
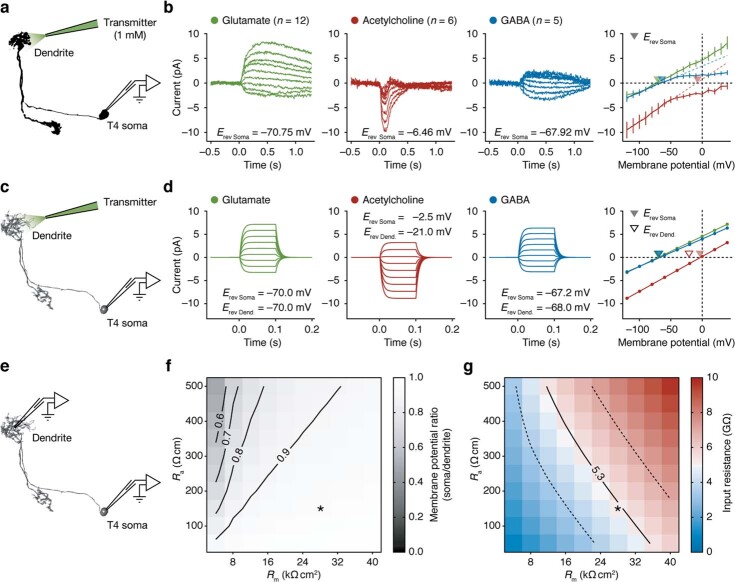
Measured and modelled T4 whole-cell currents in response to three types of neurotransmitter. **a**, Placement of pipettes for neurotransmitter application during whole-cell recording. **b**, Average whole-cell currents of T4 neurons in response to 100 ms applications of neurotransmitter to the dendrite at different holding potentials (left) and full current-voltage relationships (right). Coloured dashed lines are linear fits to measurements taken at membrane potentials in the physiologically observed range between −100 and −40 mV. Filled triangles denote reversal potentials obtained by linear fits to currents measured at the soma (*E*_rev Soma_). Data are mean ± s.e.m. *n* values indicate the number of cells. The inward rectification of GABA-induced currents could be due to coupling of GABA_B_ receptors to inwardly-rectifying potassium channels. **c**, Electron microscopic reconstruction of a T4 neuron^7^ used for compartmental modelling. Pipettes indicate approximate locations of conductances and the recording site for simulations in **d**. **d**, Somatic currents at different holding potentials simulated during 100 ms openings of conductances at the electron microscopically-determined synaptic sites corresponding to the respective transmitter (left) and current-voltage relationships (right). Conductances were adjusted in order to approximate measured reversal potentials at the soma. Filled triangles denote modelled reversal potentials at the soma (*E*_rev Soma_); open triangles denote corresponding reversal potentials at the dendritic root (*E*_rev Dend._). Note the predicted deviation of *E*_rev Soma_ from *E*_rev Dend._ for currents induced by acetylcholine, but not for currents induced by glutamate or GABA. **e**, Pipettes indicate locations of recording sites on the compartmental model (**c**) for simulations in **f**. **f**, Ratio of somatic to dendritic membrane potential in response to dendritic injection of 10 pA of depolarizing current as a function of membrane resistance (*R*_m_) and axial resistivity (*R*_a_) in the model. Note that soma and dendrite were quasi-isopotential (ratio > 0.9) across a wide range of parameters. Asterisk indicates parameter set used for simulations in **d**. **g**, Modelled somatic input resistance as a function of *R*_m_ and *R*_a_. Solid and dashed lines correspond to the measured mean input resistance ± s.d. for wild-type T4 neurons (as shown in Fig. 2h). Asterisk indicates parameter set used for simulations in **d**. Source data

**Extended Data Fig. 5:**
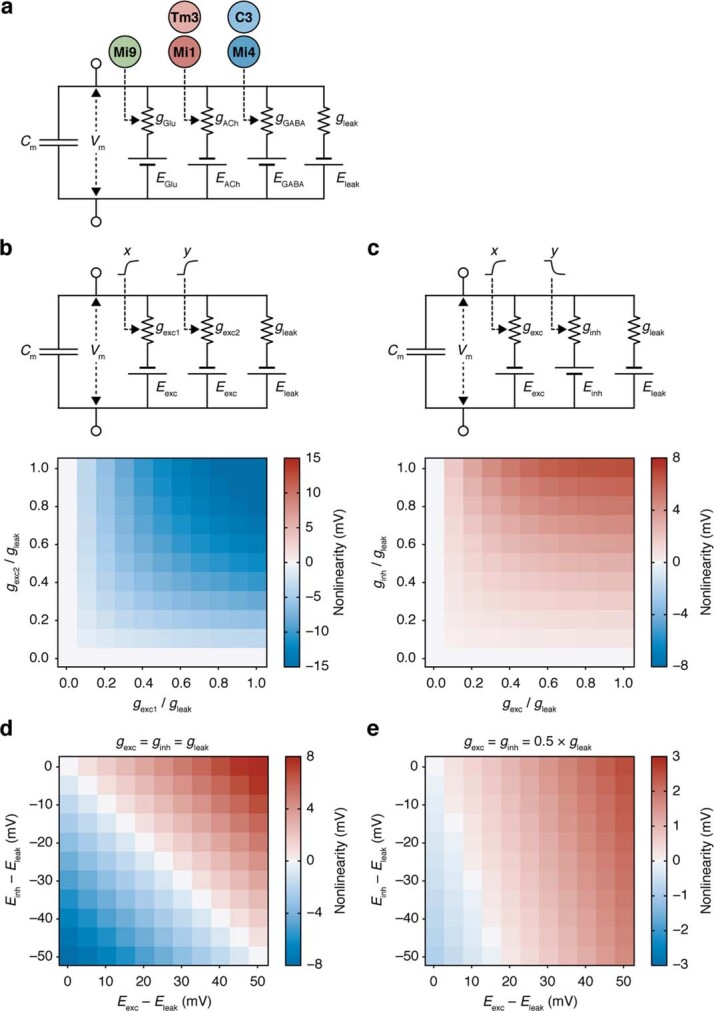
Nonlinear response properties of model circuits. **a**, Resistor-capacitor equivalent circuit of a passive T4 neuron used for simulations in Fig. 3b, c, and 5a and Extended Data Figs. 6, 7, and 8. *E*_Glu_, *E*_ACh_, and *E*_GABA_ denote the equilibrium potentials and *g*_Glu_, *g*_ACh_, and *g*_GABA_ denote the conductances associated with glutamate, acetylcholine and GABA, respectively. The signals of Mi9 neurons control *g*_Glu_, the signals of Tm3 and Mi1 neurons control *g*_Ach_, and those of C3 and Mi4 neurons act on *g*_GABA_. *V*_m_, membrane potential; *C*_m_, membrane capacitance; *g*_leak_, leak conductance. **b**, **c**, Top: Equivalent circuits of two passive isopotential neurons. One neuron (**b**) receives two input signals *x* and *y*, which control the excitatory conductances *g*_exc1_ and *g*_exc2_, respectively. The other neuron (**c**) receives one input signal *x* controlling the excitatory conductance *g*_exc_ and another input signal *y* of opposite polarity that controls the inhibitory conductance *g*_inh_. *E*_exc_, *E*_inh_, and *E*_leak_ are the equilibrium potentials of excitatory, inhibitory, and leak currents, respectively. Bottom: Nonlinearity as a function of signal amplitude for two excitatory conductances (**b**) and for one excitatory and the release from an inhibitory conductance (**c**). Nonlinearity was defined as the difference between the voltage response to both coincident inputs and the sum of the responses to each individual input. Equilibrium potentials were set to *E*_exc_ − *E*_leak_ = 50 mV and *E*_inh_ − *E*_leak_ = −10 mV. **d**, **e**, Nonlinearity of the circuit in **c** as a function of *E*_exc_ and *E*_inh_. Conductances were set to *g*_exc_ = *g*_inh_ = *g*_leak_ (**d**) or *g*_exc_ = *g*_inh_ = 0.5 × *g*_leak_ (**e**). Disinhibition supports supralinear responses over a wide range of equilibrium potentials and input signal amplitudes. Source data

**Extended Data Fig. 6:**
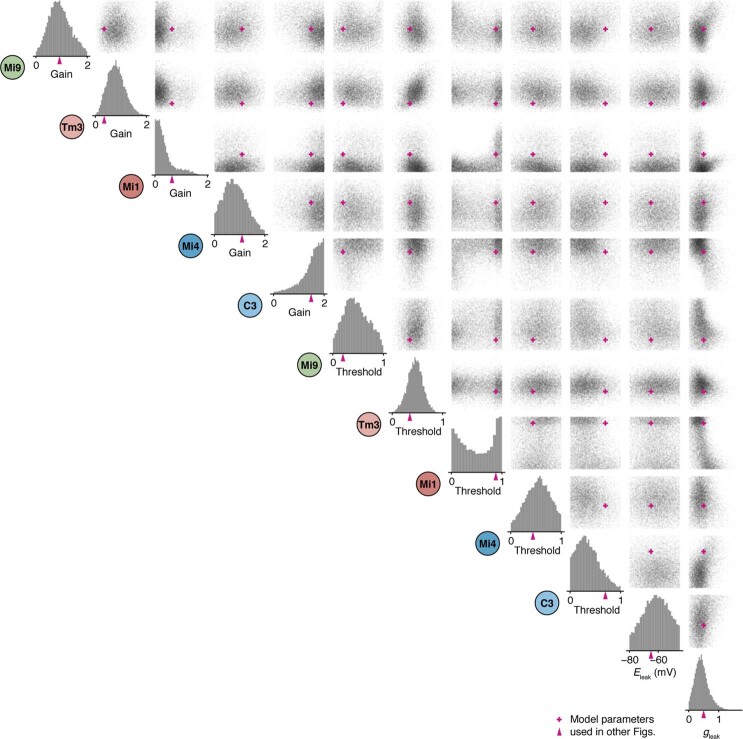
Free parameters of the conductance-based T4 neuron model. Samples (*n* = 10,000) drawn from conditional probability distributions of input neuron gains and thresholds, leak reversal potential (*E*_leak_), and leak conductance (*g*_leak_) consistent with measured voltage traces of T4 neurons inferred by deep neural density estimation^29^. Histograms of individual parameter distributions are shown at the bottom; the remaining panels each contain the relationship between two respective parameters. Pink arrowheads and crosses indicate model parameters used for simulations shown in Figs. 3b, c, and 5a and Extended Data Figs. 7b, c, and 8. Source data

**Extended Data Fig. 7:**
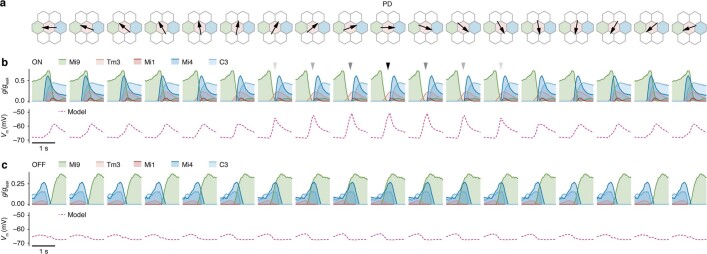
A direction-selective ‘window of opportunity’. **a**, Schematic columnar organization of T4 neuron inputs. Synapses from Mi9 neurons (green), Tm3/Mi1 neurons (red), and Mi4 /C3 neurons (blue) are each separated by one column (hexagons) resulting in direction-dependent time differences during visual motion. Arrows indicate the directions of edge motion in corresponding panels in **b** and **c**. **b**, **c**, Top: Normalized T4 cell conductances (*g*/*g*_leak_) of respective input synapses during ON (**b**) and OFF edge motion (**c**) at a velocity of 30° s^−1^ in the directions indicated in **a** using the same model parameters as in Figs. 3b, c, and 5a and Extended Data Fig. 8. Data are mean and area under curve. Arrowheads in **b** mark the coincidence of increased excitability and cholinergic excitatory input (red). Bottom: T4 cell membrane voltage (*V*_m_) responses predicted by the model. Source data

To break down the precise temporal sequences of synaptic signals evoked by visual stimulation, we obtained membrane potential recordings while moving contrast edges through the T4 neuron’s receptive field in its PD and ND (Fig. 3). Bright ON and dark OFF edges travelling at a velocity of 30° s^−1^ revealed distinct, fingerprint-like signatures of electrical activity. To explain these signatures in terms of their underlying synaptic conductances, we subjected the five columnar input elements of T4 cells to an identical set of stimuli (Fig. 3a). Our reconstructions of the receptive fields of the cells (Extended Data Fig. 1b) enabled a post hoc alignment of their responses, which we used to recreate the direction-dependent input sequences that are expected to shape the voltage responses of a T4 cell (Fig. 3b, c). With all input signals and the respective reversal potentials at hand (Extended Data Fig. 4a–d), we simulated the electrical equivalent circuit of a passive single-compartment T4 neuron (Fig. 3b, c and Extended Data Fig. 5a). Measured membrane voltages of presynaptic neurons were transformed into postsynaptic conductance values using two free parameters per neuron: a gain (that is, synaptic weight) and a threshold below which no transmission occurred. The T4 neurons’ electrically compact morphology (Extended Data Fig. 4e, f) led us to neglect the membrane capacitance. After estimating the model parameters on the basis of a least-squares fit to the average voltage responses of T4 neurons, we quantified parameter uncertainty using an artificial neural network^29^. Examination of the full range of parameter combinations compatible with our measurements confirmed the estimated values, which fell within regions of high conditional probability (Extended Data Fig. 6). In agreement with our second assumption, the voltage responses of T4 neurons to all four stimuli were captured by our passive conductance-based model (Fig. 3b, c), which naturally joins an excitatory and an inhibitory signal in a supralinear manner. While, in a passive membrane, two excitatory inputs are bound to combine sublinearly (Extended Data Fig. 5b), the coincidence of an excitatory input with the release from an inhibitory one will almost invariably yield a supralinear response^1,21^ (Extended Data Fig. 5c). Exceptions are rare and can occur only under conditions in which the reversal potential of the excitatory current is closer to the leak reversal potential than that of the inhibitory current (Extended Data Fig. 5d, e and Supplementary Equations). For ON edge motion in the PD, a brief interval of minimal inhibitory conductance—a window of opportunity^30^—opened up (Fig. 3b). The transient lack of inhibition led to the amplification of excitatory inputs from Mi1 and Tm3 neurons during the upstroke of the T4 cell’s voltage trajectory (Fig. 3b and Extended Data Fig. 7). Intuitively, this can be explained by the coincident drop in overall conductance or, in other words, the increase in input resistance.

**Fig. 4:**
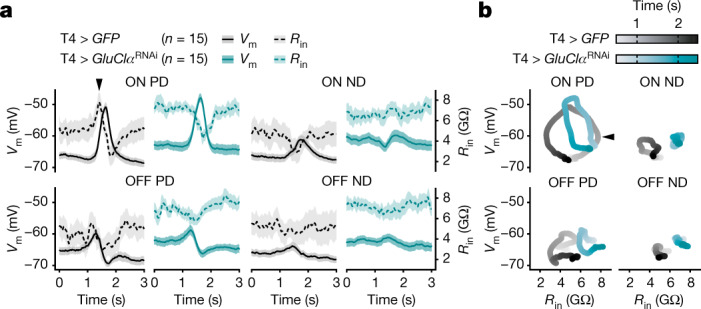
A GluClα-dependent input resistance peak. **a**, Simultaneously measured membrane potentials (*V*_m_, solid lines) and input resistances (*R*_in_, dashed lines) of T4 neurons expressing *GFP* (black) or *GFP* + *GluClα*^RNAi^ (teal) in response to ON (top) and OFF (bottom) edges moving at 30° s^−1^ in the neurons’ PD and ND. Data are mean ± s.e.m. *n* values indicate the number of cells. **b**, The average membrane potential (*V*_m_) as a function of input resistance (*R*_in_) of T4 neurons shown in **a** in response to ON (top) and OFF (bottom) edges moving in the PD (left) and ND (right). The arrowheads mark the input resistance peak. Source data.

**Extended Data Fig. 8:**
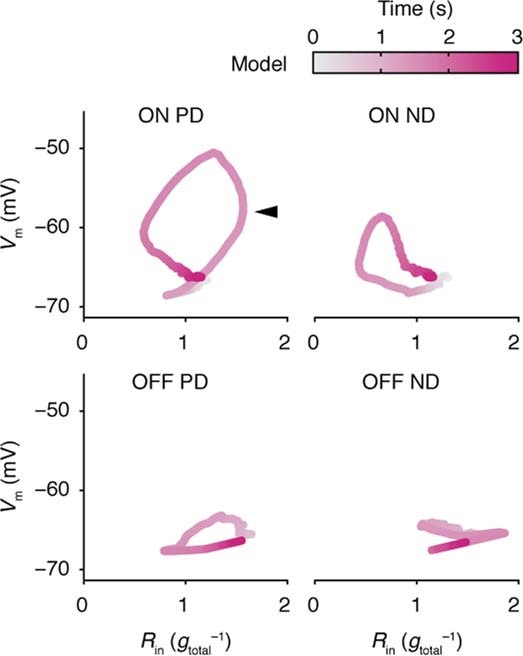
Predicted relationship between membrane potential and input resistance during edge motion. Simulated T4 cell membrane potential (*V*_m_) as a function of input resistance (*R*_in_) in response to ON (top) and OFF edges (bottom) moving at 30° s^–1^ in the preferred (PD, left) and the null direction (ND, right) of the model. The arrowhead marks the peak in input resistance. Source data

**Extended Data Fig. 9:**
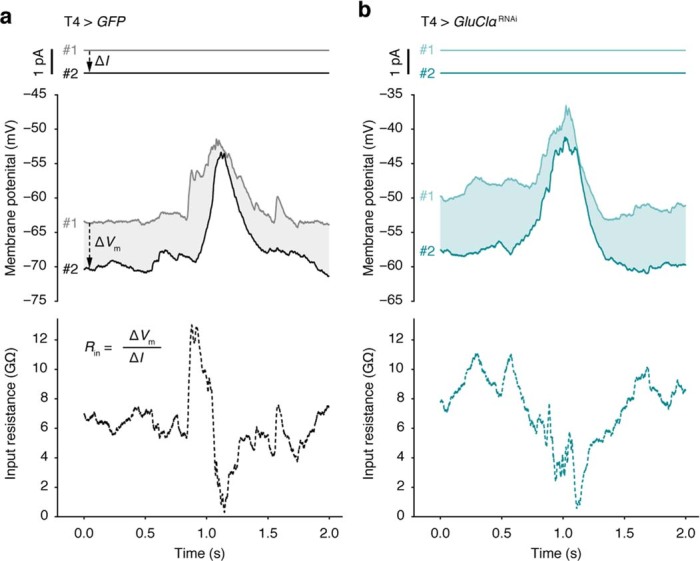
Input resistance measurements during visual stimulation. Holding currents (solid lines, top), membrane potentials (solid lines, centre), and input resistances (dashed lines, bottom) of exemplary T4 neurons expressing either *GFP* (**a**) or *GFP* + *GluClα*^RNAi^ (**b**). To obtain input resistance measurements at high temporal resolution, neurons were subjected to at least two repetitions of identical visual stimulation while recording their membrane potentials. In this case, the stimulus was an ON edge moving at 30° s^−1^ in the neuron’s preferred direction. The holding current *I* was altered in between the first (#1) and the second repetition (#2) by Δ*I* = −1 pA. The input resistance *R*_in_ at each time point was calculated as Δ*V*_m_/Δ*I*, where Δ*V*_m_ denotes the difference in membrane potential between repetitions (shaded areas/dashed arrows in **a**). Source data

Direct evidence for the predicted increase in resistance (Extended Data Fig. 8) was obtained using current-clamp experiments. We took advantage of each T4 neuron’s stereotyped responses to moving edges and presented the fly with repeated episodes of identical visual stimulation. Varying the holding current in between episodes enabled us to obtain time-locked measurements of membrane potential and resistance (Fig. 4 and Extended Data Fig. 9). For ON edges moving in the neuron’s PD, the input resistance revealed a distinct peak that preceded the depolarizing voltage excursion and amounted to approximately 147% of the initial resistance (Fig. 4). Under all other conditions, the T4 cell experienced, if anything, a dip in excitability (Fig. 4). RNAi-mediated silencing of *GluClα* pre-empted the increase in that the resistance of GluClα-deficient T4 neurons at the baseline was already equivalent to the peak values reached by wild-type neurons (Fig. 4). Owing to the shift in resting potential towards the reversal potential of acetylcholine-induced currents, depletion of GluClα also reduced the membrane potential response amplitude from 18.10 ± 0.77 mV in wild-type T4 neurons to 13.63 ± 1.05 mV in *GluClα*^RNAi^*-*expressing T4 neurons (mean ± s.e.m.; *n* = 53 and *n* = 30 cells, respectively; *P* = 0.0008, two-tailed Mann–Whitney *U*-test).

**Fig. 5:**
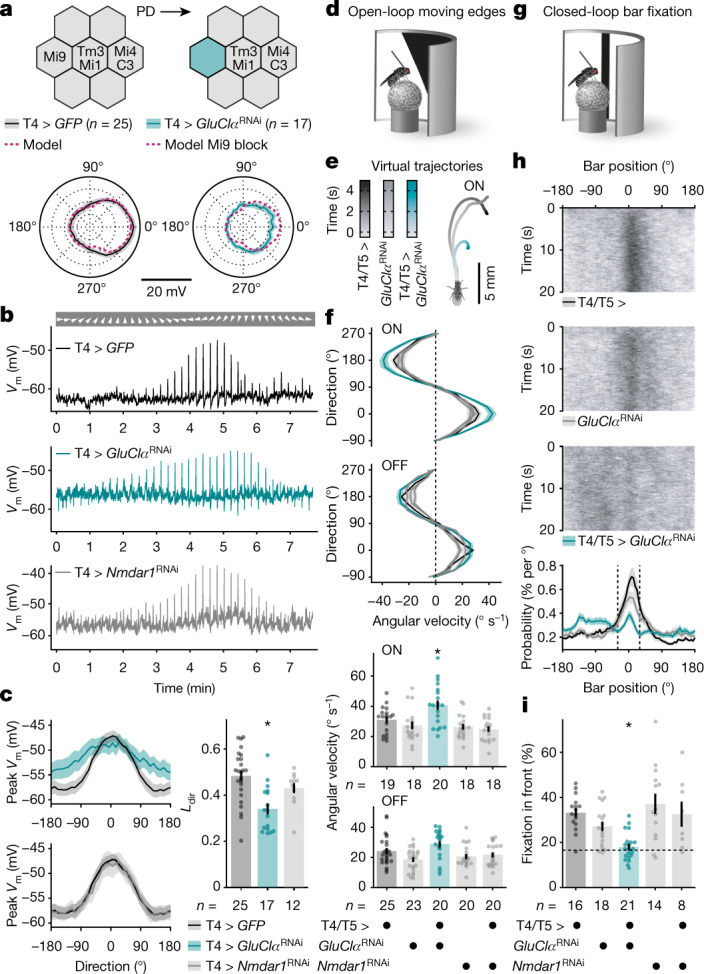
GluClα sharpens directional tuning of T4 neurons and optomotor behaviour. **a**, T4 input organization in the presence (top left) and absence of Mi9 neurons (top right). Bottom, directional tuning of T4 neurons expressing *GFP* (black) or *GFP* + *GluClα*^RNAi^ (teal) on the basis of membrane potential responses to ON edges moving at 30° s^−1^. Data are mean ± s.e.m. *n* values indicate the number of cells. The pink dashed lines show model predictions. **b**, Exemplary membrane voltage (*V*_m_) recordings from T4 neurons in **c** in response to ON edges moving in the indicated directions (arrowheads). **c**, Peak membrane voltages of T4 neurons expressing *GFP* (black), *GFP* + *GluClα*^RNAi^ (T4 > *GluClα*^RNAi^, teal) or *GFP* + *Nmdar1*^RNAi^ (T4 > *Nmdar1*^RNAi^; grey) as a function of the direction of ON edge motion (left). Data are mean ± s.e.m. Right, directional tuning (*L*_dir_) for all genotypes. Kruskal–Wallis test followed by Dunn’s multiple-comparisons test detected a significant difference of T4 > *GluClα*^RNAi^ from T4 > GFP; *P = 0.0002. The circles show individual cells. The bars show the mean ± s.e.m. *n* values indicate the number of cells. **d**, Open-loop optomotor behaviour. **e**, Average virtual walking trajectories of flies expressing *GluClα*^RNAi^ in T4/T5 cells (teal, *n* = 20) and of their parental controls (back and grey, *n* = 19 and *n* = 18, respectively) in response to ON edge motion at a 22.5° angle. **f**, The angular velocities of flies expressing *GluClα*^RNAi^ (teal) or *Nmdar1*^RNAi^ (grey) in T4/T5 neurons, and of their parental controls (black/grey), as a function of stimulus direction and polarity (top). Data are mean ± s.e.m. Bottom, absolute angular velocities scaled by horizontal stimulus components. For moving ON edges, one-way ANOVA followed by Holm–Šídák’s multiple comparisons test detected a significant difference of flies expressing *GluClα*^RNAi^ in T4/T5 cells from both parental controls; *P = 0.0105. The circles represent individual flies. The bars show the mean ± s.e.m. *n* values indicate the number of flies. **g**, Closed-loop bar fixation behaviour. **h**, Exemplary bar trajectories (832 trials and 16 flies per genotype, top) and the overall bar position probabilities (bottom) for flies expressing *GluClα*^RNAi^ in T4/T5 cells (teal) and their parental controls (back/grey). Probabilities are mean ± s.e.m. of flies in **i**. **i**, The percentage of the time that the bar occupied a 60° central window (fixation in front, dashed lines in **h**). Welch’s ANOVA followed by Dunnett’s T3 multiple comparisons test detected a significant difference of flies expressing *GluClα*^RNAi^ in T4/T5 cells from both parental controls; *P = 0.0042. The dashed line indicates the chance level. The circles represent individual flies. The bars show mean ± s.e.m. *n* values indicate the number of flies. Source data.

The ability to restrict the arithmetic repertoire of T4 neurons by interfering with the abundance of GluClα enabled us to test the prediction that multiplication sharpens directional tuning. We moved bright edges at a speed of 30° s^−1^ in 36 evenly spaced directions while recording the membrane potentials of GFP-labelled wild-type and *GluClα*^RNAi^*-*expressing T4 neurons (Fig. 5a–c). RNAi targeting transcripts of *Nmdar1*, which encodes a glutamate-gated cation channel with negligible expression in T4 cells^25–28^, was used as an additional control. Silencing *GluClα* in T4 cells in vivo replicated the effect of silencing Mi9 neurons in silico—it broadened the directional tuning curve (Fig. 5a). Response amplitudes of wild-type and *Nmdar1*^RNAi^-expressing neurons declined steeply with increasing angular distance from PD, to 72.97% and 72.74% at a deviation of 60°, respectively. The decline was much shallower in *GluClα*^RNAi^-expressing T4 neurons of which the response amplitudes at PD ± 60° still averaged 89.62% of the corresponding PD responses (Fig. 5a). Rather than enhancing voltage responses to visual motion in the PD, the presence of GluClα attenuated responses to motion in all other directions, an effect that was especially obvious at those directions not affected by inhibition from Mi4 and C3 neurons (Fig. 5a–c). This was reflected in a significant reduction of the T4 neurons’ directional tuning indices (*L*_dir_) in the absence of GluClα compared with the wild-type controls (*P* = 0.0002, Kruskal–Wallis test followed by Dunn’s multiple-comparisons test; Fig. 5c).

**Extended Data Fig. 10:**
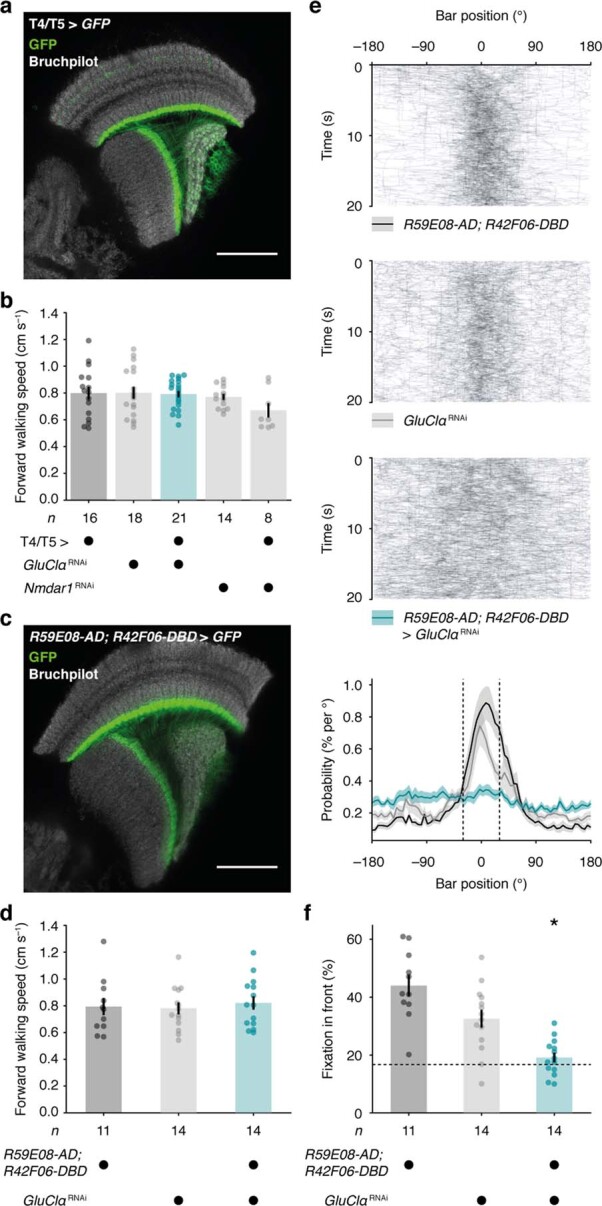
GAL4 expression patterns, walking speeds, and bar fixation. **a**, Confocal cross section through the optic lobe of a fly expressing *GFP* (green) under control of *R39H12-GAL4* (T4/T5 >) as used in behavioural experiments in Fig. 5d–i. Synaptic structures were counterstained with an antibody against bruchpilot (grey). Scale bar, 40 µm. The micrograph is representative of 8 biological replicates. **b**, Average forward walking speeds of flies expressing *GluClα*^RNAi^ (teal) or *Nmdar1*^RNAi^ (grey) in T4/T5 neurons and their parental controls (black/grey) during closed-loop bar fixation experiments in Fig. 5h, i. **c**, Confocal cross section through the optic lobe of a fly expressing *GFP* (green) under control of the split GAL4 line *R59E08-AD*; *R42F06-DBD*. Synaptic structures were counterstained with an antibody against bruchpilot (grey). Scale bar, 40 µm. The micrograph is representative of 5 biological replicates. **d**, Average forward walking speeds of flies expressing *GluClα*^RNAi^ (teal) under control of *R59E08-AD*; *R42F06-DBD* and their parental controls (black/grey) during closed-loop bar fixation in **e**, **f**. **e**, Exemplary bar trajectories (242 trials and 11 flies per genotype, top) and the overall bar position probabilities (bottom) for flies expressing *GluClα*^RNAi^ (teal) under control of *R59E08-AD*; *R42F06-DBD* and their parental controls (back/grey). Data are mean ± s.e.m. of flies in **f**. **f**, The percentage of time that the bar occupied a central 60° window (fixation in front, dashed lines in **e**). The dashed line indicates the chance level. Circles, individual flies; bars, mean ± s.e.m. Asterisk denotes a significant difference from both parental controls (*P* = 0.0012, one-way ANOVA followed by Holm–Šídák’s multiple comparisons test). *n* values indicate the number of flies. Source data

The impact of this intervention on the flies’ optomotor responses offered an opportunity to link a molecular mechanism to behavioural performance. Walking on a spherical treadmill (Fig. 5d), flies expressing *GluClα*^RNAi^ in T4 neurons and their OFF-responsive T5 twins under control of *R39H12-GAL4* (Extended Data Fig. 10a) overestimated the velocity of bright, but not of dark, edges moving in different directions. In their attempt to compensate for the perceived egomotion, animals that carried both the *GAL4* and the *UAS-GluClα*^RNAi^ transgene rotated the treadmill excessively about the vertical axis and strayed off the virtual paths of their parental controls (Fig. 5e, f). The angular velocities of animals of all other genotypes, including those expressing *Nmdar1*^RNAi^ in T4/T5 neurons, were indistinguishable (Fig. 5f). In contrast to ON-responsive T4 neurons, which are speckled with GluClα receptors at both dendritic and axonal compartments, T5 neurons feature the receptor exclusively at their axon terminals^31^. It follows that the impairment of optomotor acuity specific to moving ON edges can, in all likelihood, be attributed to a process that is localized to the dendrites of T4 neurons.

To test the ability of animals with a T4/T5-cell-restricted GluClα deficiency to hold a steady course under closed-loop conditions, we took advantage of the flies’ tendency to approach a dark vertical bar, a behaviour that depends on T4/T5 neurons^32,33^. When given the opportunity to control the bar position through their walking behaviour (Fig. 5g), control animals had a clear preference for holding the bar in front of them at 0 ± 30°. By contrast, flies expressing *GluClα*^RNAi^ in T4/T5 neurons failed to maintain a stable bearing relative to the bar (Fig. 5h) despite moving at a comparable pace (Extended Data Fig. 10b). We corroborated this discovery using another, more specific split GAL4 line (Extended Data Fig. 10c–e). Independent of the driver line used, animals with a T4/T5-cell-restricted GluClα-deficiency performed at chance level (Fig. 5i and Extended Data Fig. 10f). In accordance with our third assumption, locking T4 neurons in a state of high gain (Figs. 2h and 4) resulted in exaggerated optomotor responses (Fig. 5d–f) and impaired performance as the animals navigated a virtual environment (Fig. 5g–i). These observations reveal the behavioural significance of a multiplication-like operation in a specific type of neuron.

## Discussion

Nervous systems rely on nonlinearities to process information^1^. A multiplication-like operation—possibly the simplest form of nonlinearity—is implicated in the transformation of eye-centric into head-centric coordinates^13^, the localization of sound^12^, the combination of multisensory signals^34,35^ and the detection of visual motion^10^. The biophysical underpinnings of such an operation in a single neuron are by and large unclear. One exception is the looming detector of locusts, in which—just like on a slide rule—the sum of two logarithmically scaled signals is exponentially transformed into spike rates^36^. Other multiplicative synaptic interactions involve NMDA receptors^37,38^. Both mechanisms are contingent on threshold-like nonlinearities in the current–voltage relationships of ion channels: the gating of tetrodotoxin-sensitive sodium channels in the former and the magnesium block of NMDA receptors in the latter case. Here, we describe a multiplication-like nonlinearity that is independent of thresholds.

Using the visual circuit of the fruit fly as an example^5^, we took advantage of the neurons’ compact sizes, their known connectivity^6^ and our ability to manipulate them genetically to study the biophysical basis of the multiplication step in a Hassenstein–Reichardt detector^10^. We recorded the membrane potentials of ON motion-sensitive T4 neurons and of their columnar input elements in response to a defined set of visual stimuli. Our measurements of both pre- and postsynaptic voltages obviated the need for assumptions regarding the temporal dynamics of input signals when modelling the detector’s output. The voltage responses of T4 neurons were reproduced rather faithfully by our passive conductance-based model (Figs. 3b, c and 5a). Discrepancies between simulation and reality could be due to selective synaptic delays or the 15% of dendritic inputs from wide-field TmY15 and CT1 neurons^6,7,39^, which were not taken into account. In the model, as in our data, the supralinearity arises from the coincidence of excitation and release from shunting inhibition^23^. Such ‘multiplicative disinhibition’ constitutes the inverse operation of divisive inhibition. It is free from the voltage dependence that often limits threshold-based systems^40^ and less sensitive to changing signal amplitudes^21^ (Extended Data Fig. 5c). More broadly, theory invokes multiplication as a strategy to gate information flow^41,42^. The passive biophysical mechanism that we propose could lend itself to other systems, such as the logical conjunction of chemosensory signals^43^ or the gating of cortical afferents^44^. Motion vision in flies may provide one of many cases of multiplicative disinhibition.

## Methods

### Fly husbandry and genotypes

Flies were cultivated on a cornmeal, molasses and yeast medium under a 12 h–12 h light–dark cycle at 25 °C and 60% humidity. All of the experiments were carried out on female flies bearing at least one wild-type allele of the *white* gene. The experimenters were not blinded.

*Drosophila melanogaster* of the following genotypes were used to target transgene expression to the respective types of neuron: *P{R48A07-p65.AD}attP40*, *P{10XUAS-IVS-mCD8::GFP}su(Hw)attP5*; *P{VT046779-GAL4.DBD}attP2* was used to label Mi9 neurons, *P{R13E12-p65.AD}attP40/+*; *P{R59C10-GAL4.DBD}attP2/P{40XUAS-IVS-mCD8::GFP}attP2* was used to label Tm3 neurons, *P{R19F01-p65.AD}attP40/+*; *P{R71D01-GAL4.DBD}attP2/P{40XUAS-IVS-mCD8::GFP}attP2* was used to label Mi1 neurons, *P{R48A07-p65.AD}attP40*,* P{10XUAS-IVS-mCD8::GFP}su(Hw)attP5*;* P{R13F11-GAL4.DBD}attP2* was used to label Mi4 neurons, *P{R26H02-p65.AD}attP40/+*; *P{R29G11-GAL4.DBD}attP2/P{40XUAS-IVS-mCD8::GFP}attP2* was used to label C3 neurons and *P{R42F06-p65.AD}attP40*, *P{10XUAS-IVS-mCD8::GFP}su(Hw)attP5*; *P{VT037588-GAL4.DBD}attP2* (abbreviated T4 > *GFP*) was used to label T4 neurons, with a preference for subtypes T4c and T4d^17,27,45,46^. In electrophysiological experiments, *P{TRiP.HMC03585}attP40/P{R42F06-p65.AD}attP40*, *P{10XUAS-IVS-mCD8::GFP}su(Hw)attP5*; *P{VT037588-GAL4.DBD}attP2/+* (abbreviated T4 > *GluClα*^RNAi^) and *P{TRiP.HMS02199}attP2/P{R42F06-p65.AD}attP40*, *P{10XUAS-IVS-mCD8::GFP}su(Hw)attP5*; *P{VT037588-GAL4.DBD}attP2/+* (abbreviated T4 > *Nmdar1*^RNAi^) were used to silence the expression of *GluClα* and *Nmdar1*, respectively^47^.

In behavioural experiments, *P{UAS-Dcr-2.D}2*; *P{R39H12-GAL4}attP2* (abbreviated T4/T5 >), which yields strong and comprehensive expression in T4 and T5 neurons, was used to drive either *P{TRiP.HMC03585}attP40* (abbreviated *GluClα*^RNAi^) or *P{TRiP.HMS02199}attP2* (abbreviated *Nmdar1*^RNAi^). For the experiments in Extended Data Fig. 10c–f, *P{R59E08-p65.AD}attP40*; *P{R42F06-GAL4.DBD}attP2* was used as the driver line. All flies, including the parental controls, were heterozygous for the respective transgenes. *P{UAS-Dcr-2.D}2/P{10XUAS-IVS-mCD8::GFP}su(Hw)attP5*;* P{R39H12-GAL4}attP2/+* and *P{R59E08-p65.AD}attP40/P{10XUAS-IVS-mCD8::GFP}su(Hw)attP5*;* P{R42F06-GAL4.DBD}attP2/+* were used to visualize the expression pattern of the respective driver lines immunohistochemically.

With the exception of the strain used to label C3 (a gift from A. Nern and M. Reiser), all of the flies were obtained from the Bloomington *Drosophila* Stock Center.

### Histology

Brains of female flies (aged 1–3 days) were dissected in phosphate-buffered saline (PBS; 137 mM NaCl, 3 mM KCl, 8 mM Na_2_HPO_4_, 1.5 mM KH_2_PO_4_, pH 7.3) and fixed in 4% (w/v) paraformaldehyde in PBS overnight at 4 °C, followed by four 30 min washes in PBS containing 0.2% (v/v) Triton X-100 (PBT). To label biocytin-filled neurons, the samples were incubated with DyLight 633-conjugated streptavidin (21844, Invitrogen, 1:200) for 48 h at 4 °C, followed by four 30 min washes in PBT. To visualize GFP expression patterns driven by *R39H12-GAL4* and *R59E08-AD*;* R42F06-DBD*, brains were fixed for 25 min at room temperature and blocked in PBT containing 10% normal goat serum (NGS) overnight at 4 °C. Synaptic structures and GFP were labelled, first with mouse anti-bruchpilot antibodies (nc82, AB2314866, Developmental Studies Hybridoma Bank, 1:20) and chicken anti-GFP antibodies (600-901-215S, Rockland, 1:400), respectively, for 48 h and then with Atto 647N-conjugated goat anti-mouse IgG antibodies (610-156-040, Rockland, 1:300) and Alexa 488-conjugated goat anti-chicken IgY antibodies (A-11039, Invitrogen, 1:500), respectively, for 72 h, both diluted in PBT containing 5% NGS, at 4 °C. Immunodecorated samples were mounted in Vectashield antifade mounting medium (Vector Laboratories) and imaged on a Leica TCS SP8 confocal microscope equipped with an HCX PL APO ×63/1.30 NA glycerol-immersion objective (506353, Leica). Micrographs were acquired using the Leica Application Suite X (Leica) and processed using the Fiji distribution of ImageJ (v.2.0)^48^.

### Patch-clamp recordings

For whole-cell recordings in vivo^49,50^, female flies aged 2–24 h post-eclosion were cold-anaesthetized and fixed to a custom, laser-cut polyoxymethylene mount with soft thermoplastic wax (Agar Scientific). The preparation was submerged in extracellular solution (pH 7.3) containing 5 mM TES, 103 mM NaCl, 3 mM KCl, 26 mM NaHCO_3_, 1 mM NaH_2_PO_4_, 1.5 mM CaCl_2_, 4 mM MgCl_2_, 10 mM trehalose, 10 mM glucose and 7 mM sucrose (280 mOsM, equilibrated with 5% CO_2_ and 95% O_2_). Cuticle, adipose tissue and trachea were surgically removed in a window large enough to expose the left dorsal optic lobe. Patch pipettes (15–20 MΩ) were fabricated from borosilicate glass capillaries with outer and inner diameters of 1.5 mm and 1.17 mm or 0.86 mm, respectively, using a P-97 (Sutter Instruments) or a PC-10 (Narishige) micropipette puller. Pipettes were polished using a microforge (MF-830, Narishige) and filled with solution (pH 7.3) containing 10 mM HEPES, 140 mM potassium aspartate, 1 mM KCl, 4 mM MgATP, 0.5 mM Na_3_GTP, 1 mM EGTA and 10 mM biocytin (265 mOsM). Green fluorescent somata were targeted visually using a combination of bright-field and epifluorescence microscopy on an InVivo SliceScope (Scientifica) or an Axio Scope.A1 microscope (Zeiss), each equipped with a ×60/1.0 NA water-immersion objective (LUMPLFLN60XW, Olympus) and an LQ-HXP 120 light source (Leistungselektronik Jena). Transillumination was achieved by butt-coupling a white LED (MCWHD5, Thorlabs) to a liquid light guide, the far end of which was positioned caudally at a distance of 1 cm to the fly allowing for an unobstructed field of view. To gain access to cell membranes, a micropipette was used to make a small incision in the perineural sheath. Signals were recorded at room temperature (21–23 °C) with a MultiClamp 700B amplifier, low-pass-filtered and sampled at 10 kHz using a Digidata 1550B digitizer controlled through pCLAMP 11 software (all from Molecular Devices). Data were corrected for the liquid junction potential and analysed using custom-written software in Python v.3.7 (Python Software Foundation) using NumPy v.1.15, Pandas v.0.25, SciPy v.1.3, Matplotlib v.3.0 and pyABF v.2.1 (https://pypi.org/project/pyabf/). After temporal alignment, current-clamp data were analysed at a sampling rate of 1 kHz. The most negative membrane potential recorded within 2 min after break-in, in darkness and in the absence of a holding current was taken to represent the resting potential. Only cells with a measured resting potential that was more negative than −25 mV were characterized further. Input resistances, as plotted in Fig. 2, were calculated on the basis of linear fits to the steady-state voltage changes elicited by 1 s steps of hyperpolarizing currents (2 pA increments, starting at −10 pA). In voltage-clamp recordings, voltage steps were applied 2 s in advance of pharmacological applications and linear leak currents were subtracted.

### Visual stimulation in electrophysiological experiments

Visual stimuli were projected with two mirrors onto a cylindrical screen using two DLP Lightcrafter 3000 pico projectors (Texas Instruments) as previously described^20^. The screen covered 180° in azimuth and 105° in elevation of the fly’s left frontal visual field and doubled as a Faraday shield. Restricting the projectors to the green channel (500–600 nm) allowed for a refresh rate of 180 Hz at 8-bit colour depth and a maximal luminance of 1,274 cd m^−2^. The average luminance of stimuli, which were presented in full contrast, was set to an 8-bit greyscale value of 128 corresponding to an average luminance of ~637 cd m^−2^. Stimuli were created and predistorted to account for the curvature of the screen using the Panda3D game engine in Python v.2.7.

Receptive fields were located and characterized using a binary white-noise stimulus with a pixel size of 2.8° × 2.8°. Samples were drawn at a rate of 60 Hz and projected onto the screen for durations ranging from 3 min to 20 min. Stimuli and simultaneously recorded membrane potentials were time-locked using a continuously recorded trigger signal on the screen. Stimulus files were exported after lossless compression and cross-correlated to each neuron’s recorded membrane voltage using standard techniques for reverse correlation in Python (v.3.7)^20^. Slow voltage drifts were corrected post hoc by subtracting a low-pass-filtered version of the signal obtained using a Gaussian blur with a standard deviation of 60 s. The reverse correlation was calculated as

$$K(x,\tau )={\int }_{0}^{T}{\rm{d}}tS(x,t-\tau )\times {V}_{{\rm{m}}}(t),$$

where *V*_m_ denotes the neuron’s baseline-subtracted membrane voltage at time point *t* and *S* denotes the stimulus at position *x* and time point *t − τ* for values of *τ* ranging from −0.5 to +3.0 s. The resulting spatiotemporal receptive fields were converted into standard scores. Only neurons with clear standard score peaks (typically >4 s.d. from the mean) and with receptive field centres >8 px (22.48°) from the bezel of the screen were included in the analysis to guarantee full coverage of the surround. Receptive fields were normalized and aligned in space using the extremum (that is, the maximum or minimum with the highest absolute value) of the standard score as a point of reference, which was placed at 0°. After cropping the individual spatial receptive fields to the largest common region holding data from all neurons, scores were averaged across neurons of one class. For Fig. 1, averages were upsampled by a factor of 10 by linear interpolation and smoothed with a Gaussian filter (1.8 px s.d.). For direction-selective T4 neurons, individual receptive fields were rotated in space to align along the neurons’ PDs; therefore, in Fig. 1e, azimuth and elevation do not necessarily correspond to horizontal and vertical coordinates on the screen, but to coordinates parallel and orthogonal to the T4 cell’s PD.

To determine a neuron’s PD, square-wave gratings with a spatial wavelength of 30° spanning the full extent of the screen were moved at a temporal frequency of 1 Hz in eight different directions separated by 45°. The neuron’s peak membrane voltage during motion, after subtracting a 1 s prestimulus baseline, was taken to represent the magnitude of a Euclidean vector **v**(*φ*) pointing in the direction given by the angle of rotation *φ* of the associated stimulus. PD was defined as the direction of the resultant of all individual vectors. Temporal frequency tuning curves were measured using gratings of the above properties that were moved alternatingly in PD and ND (that is, PD + 180 ) at temporal frequencies ranging from 0.5 Hz to 16.0 Hz. Δ*V*_m_ was defined as the absolute difference between the maximal and minimal membrane potential.

The fine-grained directional tuning curves in Fig. 5 were assessed using ON edges moving at 30° s^−1^ in 36 evenly spaced directions. Membrane potentials were recorded in the presence of a constant holding current of −1 pA, which enabled stable recordings over extended periods of time. In Fig. 5c, |**v**(*φ*)| was defined as the maximum of a Voigt profile fit to the membrane potential in a 700 ms time window surrounding the peak response during motion in the respective direction using the VoigtModel function of the lmfit.models module in Python v.3.7. Thus the readout incorporated more data points than just the maxima of the raw traces. To make directional tuning curves comparable between experiments and genotypes, each neuron’s PD was aligned post hoc to 0° and its tuning curve was minimum–maximum normalized. Directional tuning was quantified as the magnitude of the resultant vector divided by the sum of the individual vectors’ magnitudes:

$${L}_{{\rm{dir}}}=\,|\frac{{\Sigma }_{\phi }{\bf{v}}(\phi )}{{\Sigma }_{\phi }|{\bf{v}}(\phi )|}|$$

For the experiments in Fig. 3, bright (ON) and dark (OFF) edges were moved across the screen at a velocity of 30° s^−1^. The responses of individual neurons of one type were temporally aligned based on the cross-correlation maximum between the time derivative of the low-pass-filtered membrane potential of each neuron and that of one hand-picked template neuron in response to ON edges (moving in PD for T4 cells). The responses of different input neuron classes were aligned based on the relative distances of the template neurons’ receptive field centres on the screen. Correct alignment was verified by recording light intensities from a 5°-wide area of the screen located at the respective template neuron’s receptive field centre using a custom-built photodiode under identical stimulus conditions.

Time-locked measurements of a T4 neuron’s membrane potential and input resistance (Fig. 4 and Extended Data Fig. 9) were achieved through repeated presentations of identical stimuli with varying holding current amplitudes ranging from −5 to 0 pA. The slope of a linear regression of voltages onto holding currents provided a measure of the neuron’s input resistance at each time point. For experiments with only two different holding current amplitudes, the slope of the regression is equivalent to the input resistance calculated as Δ*V*_m_/Δ*I*, where Δ*V*_m_ denotes the change in membrane potential and Δ*I* denotes the change in holding current in between repetitions. Resistances shown in Fig. 4 were smoothed with a Gaussian filter (13 ms s.d.). Input resistances did not change significantly throughout recording sessions. The difference in input resistance between the start and the end of recording sessions averaged at 0.28 ± 0.56 GΩ (mean ± s.e.m., *n* = 30 cells; *P* = 0.6143, two-tailed paired Student’s *t*-test).

### Pharmacology

For applications of glutamate, acetylcholine and GABA, a micropipette with a bore diameter of 5 µm was filled with 1 mM of neurotransmitter (dissolved in extracellular solution) and aimed at the GFP-labelled T4 dendrites in layer 10 of the medulla. To elicit transient neurotransmitter responses in patch-clamped T4 neurons, pressure (50 kPa) was applied in 100 ms pulses using a PDES-02DX pneumatic drug ejection system (NPI Electronic). For long-lasting responses during input resistance measurements, pulse times were increased to 500 ms. Two wild-type neurons were lost after the third glutamate application during patch-clamp recordings for Fig. 2e and were excluded from the repeated-measures analysis.

### Multi-compartment model

We built a passive compartmental model of a T4 neuron (Extended Data Fig. 4c, d) in Python v.3.7 to account for possible space-clamp problems due to neuronal morphology in voltage-clamp experiments and to assess signal propagation between dendrite and soma (Extended Data Fig. 4e, f). The model was based on an electron microscopic reconstruction^7^ (http://neuromorpho.org/neuron_info.jsp?neuron_name=T4a-25_85) and comprised 2,012 compartments. A connectivity matrix, which held values of 1 where two compartments were connected and values of 0 otherwise, was used as a template to calculate a conductance matrix *M*. The latter was based on the three-dimensional coordinates and the length as well as the diameter of each compartment assuming, unless stated otherwise, an axial resistivity (*R*_a_) of 150 Ω cm, a membrane resistance (*R*_m_) of 28 kΩ cm^2^, and a specific membrane capacitance (*C*_m_) of 1 µF cm^−2^. All parameters were on the same scale as those commonly used to model *Drosophila* neurons^51^ and were considered to be uniform across the entire cell. Varying *R*_a_ and *R*_m_ over a biophysically plausible range had negligible effects on model output (Extended Data Fig. 4f, g).

The voltage vector **V**_m_(*t*) indicating the membrane potential of each compartment and at each time point *t* was determined by using the sparse.linalg.spsolve function of the SciPy v.1.3 module to iteratively solve the matrix equation *M* × **V**_m_(*t*) = **V**_m_(*t − *1) × **c**_m_/Δ*t + E*_leak_ × **g**_leak_ + **I**(*t*), where **V**_m_(*t − *1) denotes the voltage vector at the previous time point, **c**_m_ is the vector holding the specific capacitances of all compartments, Δ*t* denotes the time step, *E*_leak_ denotes the leak reversal potential, **g**_leak_ denotes the vector holding the specific transmembrane leak conductances of all compartments and **I**(*t*) is the vector indicating the current injected at time point *t* into each compartment. Simulations were performed with a fixed Δ*t* of 0.1 ms. If only steady-state was considered, the diagonal of the conductance matrix *M* held no capacitive conductances and the right side of the equation simplified to *E*_leak_ × **g**_leak_ + **I**(*t*). At the time of transmitter application, synaptic conductances were added both to the diagonal of the conductance matrix and, multiplied by the reversal potential of the current, to the right side of the equation.

To simulate voltage clamp, the current injected at the soma was calculated on the basis of the difference between the chosen command voltage *V*_cmd_ and the actual potential at the soma *V*_m,soma_ using a proportional-integral control loop that served to emulate a voltage-clamp amplifier in Python v.3.7. The current to be injected at time point *t* was calculated as *I*(*t*) = *K*_p_ × (*V*_cmd_(*t*) − *V*_m,soma_(*t*)) + *K*_i _× *I*(*t *− 1); where *K*_p_ denotes the proportional gain and *K*_i_ the integral gain. With values of 2 × 10^9^ and 1 for *K*_p_ and *K*_i_, respectively, *V*_m,soma_ could be clamped reliably at the desired *V*_cmd_ under all conditions and synaptic inputs.

### Single-compartment model

Recorded membrane voltages of input neurons were averaged, minimum–maximum normalized (retaining the signal ratios across stimuli) and converted into relative conductances using a rectilinear transfer function with two free parameters per neuron: a threshold below which all conductances were set to 0 and a gain (that is, a scaling factor). Taking into account an average inter-ommatidial angle *θ* of 4.8° (refs. ^52,53^) and the edge velocity *v* of 30° s^−1^, conductances of Mi9 neurons and those of Mi4 and C3 neurons were advanced or delayed in time, respectively, by Δ*t* relative to those of Mi1 and Tm3 neurons, depending on the angle *φ* of the virtual edge: Δ*t* = *θ *cos*φ/v*.

For each stimulus condition, the membrane potential of the T4 neuron was calculated as

$${V}_{m}=\,\frac{{E}_{{\rm{Glu}}}\,{g}_{{\rm{Mi9}}}+{E}_{{\rm{ACh}}}\,({g}_{{\rm{Tm3}}}+{g}_{{\rm{Mi1}}})+{E}_{{\rm{GABA}}}\,({g}_{{\rm{Mi4}}}+{g}_{{\rm{C3}}})+\,{E}_{{\rm{leak}}}{g}_{{\rm{leak}}}}{{g}_{{\rm{Mi9}}}+{g}_{{\rm{Tm3}}}+\,{g}_{{\rm{Mi1}}}+\,{g}_{{\rm{Mi4}}}+{g}_{{\rm{C3}}}+{g}_{{\rm{leak}}}},$$

where *g* denotes the relative conductance associated with each input neuron and *E* denotes the reversal potential of the respective synaptic current with *E*_Glu_ = −71 mV, *E*_ACh_ = −21 mV and *E*_GABA_ = −68 mV as measured/modelled in voltage-clamp experiments (Extended Data Fig. 4a–d). Owing to the compact size of a T4 neuron, the small amplitudes of capacitive currents (in relation to the steady-state amplitudes) and their short time constants (in relation to those of synaptic currents) eliminated the need for a differential equation to calculate *V*_m_. Free parameters (thresholds, gains, *E*_leak_ and *g*_leak_) were estimated from a least-squares fit to measured membrane voltage traces of T4 neurons, computed with the help of the optimize.minimize function of the SciPy v.1.3 module and hand-tuned using a FaderPort 16-channel mix production controller (Presonus). Upper and lower bounds for parameter values were set to 0 and 1 for thresholds, 0 and 2 for gains, −80 mV and −45 mV for *E*_leak_, and 0 and 3 for *g*_leak_, respectively. The parameters used for the simulations shown in Figs. 3b, c and 5a and Extended Data Figs. 7b, c and 8 were as follows: Mi9_gain_ = 0.92, Tm3_gain_ = 0.35, Mi1_gain_ = 0.65, Mi4_gain_ = 1.10, C3_gain_ = 1.49, Mi9_thld_ = 0.20, Tm3_thld_ = 0.35, Mi1_thld_ = 0.88, Mi4_thld_ = 0.44, C3_thld_ = 0.70, *E*_leak_ = −65.0 mV and *g*_leak_ = 0.50, where ‘thld’ refers to the respective threshold values.

To validate our choice of parameters and to quantify the sensitivity, robustness and uniqueness of parameter sets, we resorted to simulation-based inference^29^, which enabled us to examine the full range of possible parameter combinations. We used 20,000 model simulations, drawing parameters from uniform distributions within the above bounds, to train the artificial neural network implemented in the sequential neural posterior estimation (SNPE) algorithm of the software package sbi (v.0.8)^54^. On the basis of Bayesian inference, SNPE provided a conditional probability distribution *P*(*α*|*V*_data_), which is high for parameter sets *α* that are consistent with the experimentally measured voltage traces *V*_data_, but close to zero otherwise. To visualize *P*(*α*|*V*_data_) we drew 10,000 sample parameter sets that are compatible with *V*_data_ and compared them to our chosen parameters (Extended Data Fig. 6). All of the simulations were written in Python v.3.7.

### Behaviour

Female flies (aged 1–5 days) were cold-immobilized and attached to a pin with light-curing composite glue (Sinfony Opaque Dentin, 3M) using dental curing light (440 nm, New Woodpecker). Five independent locomotion recorders^32^ were operated in parallel. In each recorder, a tethered fly was positioned on top of an air-suspended polyurethane sphere with a diameter of 6 mm and a weight of around 40 mg. The sphere floated freely on an air stream supplied by a rotary vane pump (G6/01-K-EB9L, Gardner Denver Thomas) through an inlet at the bottom of a concave holder, allowing the walking fly to rotate the sphere about any axis through its centre. The rotation of the spherical treadmill, lit by an infrared LED (JET-800-10, Roithner Electronics), was tracked at 4 kHz and digitized at 200 Hz using a custom-designed system based on two optical computer mouse sensors focused on two 1 mm^2^ equatorial squares at ±30° from the centre of the sphere^55^. A camera (GRAS-20S4M-C, Point Grey Research) was used to facilitate proper positioning of the fly on the ball. To encourage prolonged walking, the air temperature surrounding the fly was maintained at 34 ± 0.1 °C using a custom-built air conditioning system with a Peltier heater (QC-127-1.4-6.0MS, Quick-Cool) and a thermometer positioned below the sphere.

Visual stimuli were presented with a refresh rate of 120 Hz on three liquid crystal displays (2233RZ, Samsung) arranged vertically to form a U-shaped visual arena surrounding the fly, which spanned approximately 270° in azimuth and 120° in elevation of the fly’s visual field at a resolution of <0.1°. The maximal luminance of the displays was 131 cd m^−2^; the average intensity of stimuli, which were presented at a Michelson contrast of 50%, was set to an 8-bit greyscale value of 100. Stimuli were created, and predistorted to mimic a cylindrical panorama, using the Panda3D game engine in Python v.2.7.

In open-loop experiments, ON and OFF edges were moved at a velocity of 60° s^−1^ in 16 evenly spaced directions. Owing to the geometry of the visual arena, full translation of edges at different angles required variable amounts of time. Thus, to limit stimulus durations to 5 s, an edge of which the direction of motion deviated from the cardinal directions was initialized with a small segment of the edge already present in one of the outer corners (never covering any part of the central display). Edges started moving 0.5 s after stimulus initialization and crossed the arena within 5 s. In a single experiment (~80 min), flies experienced 50 trials of either ON or OFF edges moving in all 16 directions in a pseudorandom order. The first 15 trials were used to equilibrate the temperature and to accustom the fly to the treadmill and were excluded from analyses. As inclusion criteria, we used a forward walking speed of ≥0.15 cm s^−1^ on a trial-by-trial basis and a minimum of ten trials per fly. To correct for a possible constant turning bias, the time-averaged rotational velocity of each full trial (comprising all 16 directions) was subtracted from all measurements of the corresponding trial. The optomotor response was quantified as the average rotational velocity during 5 s of edge motion in the corresponding direction. The slope of a linear regression of optomotor responses onto the absolute horizontal stimulus components |cos*φ*| served as a single measure of an animal’s angular velocity across different edge angles *φ*.

In closed-loop experiments, bar-fixation was assessed using a 10°-wide dark vertical bar, the position of which along the azimuth was controlled in real time by the rotation of the spherical treadmill (Δbar position = −rotation about *z* axis, updated every ~9 ms). The bar appeared at a random position between −180° and 180° at the start of each 20 s trial, during which the fly could control the bar’s position through its walking behaviour. One experiment (~60 min) consisted of 180 trials, the first 40 of which were not analysed, as they served to equilibrate the temperature and to accustom the fly to the virtual environment. For the results presented in Extended Data Fig. 10d–f, each experiment consisted of 80 longer multi-stimulus trials, the first 10 of which were excluded. Only trials with a forward walking velocity of ≥0.40 cm s^−1^ and flies with at least 50 (20 for Extended Data Fig. 10d–f) of such trials were included in the analysis. To avoid possible turning bias (for example, due to skewed mounting), flies whose average turning deviated from zero by >10° s^−1^ were excluded. Probability density functions of bar positions were calculated for each 20 s trial using a bin width of 5° before averaging over trials. The measure ‘fixation in front’ was obtained by summing the probabilities of finding the bar in a 60° window in front of the fly and averaging these probabilities over trials.

### Statistics and reproducibility

Statistical tests were performed in Prism v.9.2 (GraphPad). Details, including test statistics, degrees of freedom and exact *P* values for statistical analyses of data shown in Figs. 2 and 5 and Extended Data Fig. 10 are reported in Supplementary Tables 1 and 2.

Data were assessed for normality and equality of variances using Shapiro–Wilk and Brown–Forsythe tests, respectively. Two groups of normally distributed data were compared using two-tailed Student’s *t*-tests (paired if applicable). Two groups of nonparametric data were compared using two-tailed Mann–Whitney *U*-tests for independent datasets and using Wilcoxon matched-pairs signed-ranks test for paired datasets. Differences between the means of multiple independent groups of data that met the assumptions of normality and equality of variances were compared using one-way ANOVA followed by Holm–Šídák’s multiple-comparisons test. Where the assumptions of normality or of equality of variances were violated, group means were compared using Kruskal–Wallis tests followed by Dunn’s multiple-comparisons test or by Welch’s ANOVA followed by Dunnett’s T3 multiple-comparisons test, respectively. Reported *P* values were corrected for multiple comparisons. Data shown in Fig. 2e were analysed using two-way repeated measures ANOVA with Geisser–Greenhouse correction. For multiple comparisons with parental controls, the highest of two* P* values was reported in the figure legend.

No sample size calculations were performed before experimentation. Sample sizes were chosen to match or exceed standard sample sizes in the field. Sample sizes in electrophysiological experiments correspond to the number of cells, each of which was recorded in a different animal. Sample sizes in behavioural experiments correspond to the number of flies. The investigators were not blinded. Randomization was not applicable, because flies were grouped on the basis of genotype. In open-loop behavioural experiments (Fig. 5d–f) and all experiments involving two directions of visual stimuli, stimulus directions were alternated randomly; all of the remaining visual stimuli were presented in a strict sequence to enable quick, intuitive interpretation (Figs. 1f and 5b). Two wild-type neurons were lost after the third glutamate application during patch-clamp recordings for Fig. 2e and were excluded from the repeated-measures analysis. Six cells were lost during voltage-clamp experiments shown in Fig. 2f and Extended Data Fig. 4b due to pneumatic ejection. The current–voltage relationships of those cells do not include all, but at least six, data points per cell.

### Reporting summary

Further information on research design is available in the Nature Research Reporting Summary linked to this paper.

## Online content

Any methods, additional references, Nature Research reporting summaries, source data, extended data, supplementary information, acknowledgements, peer review information; details of author contributions and competing interests; and statements of data and code availability are available at 10.1038/s41586-022-04428-3.

## Supplementary information

Supplementary Information This file contains Supplementary Equations and Supplementary Tables 1 and 2.

Reporting Summary

## Data Availability

Data are available at the Edmond Open Research Data Repository of the Max Planck Society (10.17617/3.8g). Source data are provided with this paper.

## Source data

Source Data Fig. 1
Source Data Fig. 2
Source Data Fig. 3
Source Data Fig. 4
Source Data Fig. 5
Source Data Extended Data Fig. 1
Source Data Extended Data Fig. 2
Source Data Extended Data Fig. 4
Source Data Extended Data Fig. 5
Source Data Extended Data Fig. 6
Source Data Extended Data Fig. 7
Source Data Extended Data Fig. 8
Source Data Extended Data Fig. 9
Source Data Extended Data Fig. 10

## Code availability

Custom-written code is available at the Edmond Open Research Data Repository of the Max Planck Society (10.17617/3.8g).
