# Supplementary material for: A biophysical account of multiplication by a single neuron
Source: Nature. 2022 Feb 23;603(7899):119–23. doi: 10.1038/s41586-022-04428-3 (PMC8891015; doi:10.1038/s41586-022-04428-3)
Supplement: Supplementary file 1 — This file contains Supplementary Equations and Supplementary Tables 1 and 2. [file 41586_2022_4428_MOESM1_ESM.pdf]

nature portfolio

https://doi.org/10.1038/s41586-022-04428-3

Supplementary information

A biophysical account of multiplication by a single neuron

In the format provided by the authors and unedited

Supplementary information

Supplementary equations

Here, we examine under which conditions a passive membrane can give rise to multiplication-like signal amplification. To extract the nonlinearity, we compare the response to two coincident inputs with the sum of the responses to each individual input presented in temporal isolation ('linear expectation'). We consider the simple case of an electrical equivalent circuit of a passive isopotential neuron that receives two excitatory input signals x and y, which control the excitatory conductances g_{exc1} and g_{exc2}, respectively (Extended Data Fig. 5b). The neuron's membrane potential V_m at steady state is given by

$$V_m = \frac{E_{exc}\,(g_{exc1} + g_{exc2}) + E_{leak}\,g_{leak}}{g_{exc1} + g_{exc2} + g_{leak}}\,;$$

where E_{exc} and E_{leak} are the reversal potentials of excitatory and leak currents, respectively, and g_{leak} is the leak conductance. In the absence of input signals (i.e. when $x = y = 0$), the neuron's resting potential $V_{rest} = E_{leak}$.

If we express the membrane potential response ΔV as the difference between V_m and V_{rest} and all conductances relative to g_{leak}, then the membrane potential response to two coincident excitatory inputs is

$$\Delta V = \frac{E_{exc}(g_{exc1} + g_{exc2}) + E_{leak}}{g_{exc1} + g_{exc2} + 1} - V_{rest}\;.$$

For $g_{exc1} = x$, $g_{exc2} = y$, and $V_{rest} = E_{leak} = 0$ the response to the combined inputs can be written as

$$\Delta V_{1,2} = E_{exc}\frac{x + y}{x + y + 1}\,.$$

The individual responses ΔV_1 and ΔV_2 to each input presented in isolation are

$$\Delta V_1 = E_{exc}\frac{x}{x + 1} \text{ and } \Delta V_2 = E_{exc}\frac{y}{y + 1}\,.$$

Now we show that, for two excitatory inputs, $\Delta V_{1,2}$ is always smaller than the linear expectation $\Delta V_1 + \Delta V_2$:

$$E_{exc}\frac{x+y}{x+y+1} < E_{exc}\frac{x}{x+1} + E_{exc}\frac{y}{y+1}\,.$$

Factoring out E_{exc}, we obtain

$$\frac{x+y}{x+y+1} < \frac{x}{x+1} + \frac{y}{y+1}\,.$$

The left expression can be broken into two components:

$$\frac{x}{x+y+1} + \frac{y}{x+y+1} < \frac{x}{x+1} + \frac{y}{y+1}\,.$$

If follows that, for positive non-zero values of x and y,

$$\frac{x}{x+y+1} < \frac{x}{x+1} \text{ and } \frac{y}{x+y+1} < \frac{y}{y+1} .$$

If $a < c$ and $b < d$, then $a + b < c + d$. Therefore, the response of a passive neuron to two coincident excitatory inputs $\Delta V_{1,2}$ is always sublinear; i.e. smaller than the linear expectation $\Delta V_1 + \Delta V_2$ (Extended Data Fig. 5b).

Next, we consider the pairing of an excitatory with an inhibitory input (Extended Data Fig. 5c). This neuron's steady-state membrane potential is

$$V_m = \frac{E_{exc}g_{exc}+E_{inh}g_{inh}+E_{leak}g_{leak}}{g_{exc}+g_{inh}+g_{leak}} .$$

As before, we let $g_{exc} = x$, but the inhibitory conductance g_{inh} follows $1 - y$, meaning that it decreases with increasing signal y (just like Mi9 neurons hyperpolarize with increasing light intensity). Again, we express the membrane potential response ΔV as the difference between V_m and V_{rest} and all conductances relative to g_{leak}:

$$V_m = \frac{E_{exc}\, x + E_{inh}\,(1-y)+E_{leak}}{x + (1-y) + 1} \text{ and}$$

$$\Delta V = V_m - V_{rest} .$$

All reversal potentials are expressed as the difference to E_{leak}, which we set to zero ($E_{leak} = 0$). Note that, unlike before, the neuron's membrane potential at rest (i.e. when $x = y = 0$) is now $V_{rest} = E_{inh}/2$. The response to the combined inputs is

$$\Delta V_{1,2} = \frac{E_{exc}\, x + E_{inh}\,(1-y)}{x - y + 2} - \frac{E_{inh}}{2} ;$$

which can be written as

$$\Delta V_{1,2} = \frac{x\,(2E_{exc} - E_{inh}) - yE_{inh}}{2(2 + x - y)} .$$

The individual responses are

$$\Delta V_1 = \frac{x(2E_{exc} - E_{inh})}{2(2 + x)} \text{ and } \Delta V_2 = \frac{-yE_{inh}}{2(2 - y)} .$$

In the following, we show under which conditions, $\Delta V_{1,2}$ is larger than the linear expectation $\Delta V_1 + \Delta V_2$:

$$\frac{x(2E_{exc} - E_{inh}) - yE_{inh}}{2(2 + x - y)} > \frac{x(2E_{exc} - E_{inh})}{2(2 + x)} - \frac{yE_{inh}}{2(2 - y)}.$$

This simplifies to

$$\frac{x(2E_{exc} - E_{inh}) - yE_{inh}}{2 + x - y} > \frac{x(2E_{exc} - E_{inh})}{2 + x} - \frac{yE_{inh}}{2 - y} .$$

Put over a common denominator, it can be written as

$$(x(2E_{exc} - E_{inh}) - yE_{inh})(2 + x)(2 - y) \; > \; x(2E_{exc} - E_{inh})(2 + x - y)(2 - y) - yE_{inh}(2 + x - y)(2 + x) .$$

Expansion leads to

$$x(2E_{exc} - E_{inh})(2 + x)(2 - y) - yE_{inh}(2 + x)(2 - y) > x(2E_{exc} - E_{inh})(2 + x)(2 - y) - xy(2E_{exc} - E_{inh})(2 - y) - yE_{inh}(2 - y)(2 + x) - xyE_{inh}(2 + x) .$$

Subtraction of the blue and the red expressions on both sides yields

$$0 > -xy(2E_{exc} - E_{inh})(2 - y) - xyE_{inh}(2 + x) .$$

Division by $(-xy)$ reverses the inequality sign:

$$(2E_{exc} - E_{inh})(2 - y) + E_{inh}(2 + x) > 0 .$$

This simplifies to

$$2E_{exc}(2 - y) + E_{inh}(y + x) > 0 ;$$

or

$$E_{exc} > -E_{inh}\frac{x+y}{2(2-y)} .$$

Note that E_{exc} and E_{inh} are expressed as the difference to E_{leak}. For $0 \leq x \leq 1$ and $0 \leq y \leq 1$ (i.e. positive conductances smaller or equal to g_{leak}) and $|E_{exc}| > |E_{inh}|$, the above inequality always holds. In the extreme case of $x = y = 1$ the coincidence of an excitatory input with the release from an inhibitory one gives rise to a supralinearity as long as E_{inh} is closer to E_{leak} than E_{exc} (Extended Data Fig. 5d). Other values of x and y yield supralinear responses over much wider ranges of E_{exc} and E_{inh} (Extended Data Fig. 5e).

Supplementary Table 1. Statistical analyses of Figs. 2, 5.

Figure	Statistical test	Measured variable	Experimental groups/comparisons	Test statistic	*P*
**2c**	Shapiro–Wilk test	Membrane potential change	T4 > *GFP*	$W = 0.9317$	0.0849
	Shapiro–Wilk test	Membrane potential change	T4 > *GluClα*^{RNAi}	$W = 0.8429$	0.0178
	Two-tailed paired Student's *t*-test	Membrane potential	T4 > *GFP* before **vs.** after glutamate	$t_{25} = 6.124$	2.111×10^{-6}
	Two-tailed Wilcoxon matched-pairs signed rank test	Membrane potential	T4 > *GluClα*^{RNAi} before **vs.** after glutamate	$W = 27.00$	0.4263
**2e**	Two-way repeated-measures ANOVA	Input resistance	Genotype × glutamate	$F_{8, 216} = 9.743$	1.579×10^{-11}
			Genotype	$F_{1, 27} = 2.263$	0.1441
			Glutamate	$F_{3.515, 94.92} = 22.57$	3.458×10^{-12}
			Cell	$F_{27, 216} = 77.93$	4.295×10^{-96}
**2g**	Shapiro–Wilk test	Resting membrane potential	T4 > *GFP*	$W = 0.9827$	0.0178
	Shapiro–Wilk test	Resting membrane potential	T4 > *GFP, GluClα*^{RNAi}	$W = 0.9915$	0.7673
	Two-tailed Mann–Whitney *U* test	Resting membrane potential	T4 > *GFP* **vs.** T4 > *GFP, GluClα*^{RNAi}	$U = 2959$	3.404×10^{-23}
**2h**	Shapiro–Wilk test	Input resistance	T4 > *GFP*	$W = 0.9708$	0.0002
	Shapiro–Wilk test	Input resistance	T4 > *GFP, GluClα*^{RNAi}	$W = 0.9677$	0.0115
	Two-tailed Mann–Whitney *U* test	Input resistance	T4 > *GFP* **vs.** T4 > *GFP, GluClα*^{RNAi}	$U = 5979$	4.751×10^{-11}
**5c**	Shapiro–Wilk test	L_{dir}	T4 > *GFP*	$W = 0.9626$	0.4679
	Shapiro–Wilk test	L_{dir}	T4 > *GluClα*^{RNAi}	$W = 0.8984$	0.0640
	Shapiro–Wilk test	L_{dir}	T4 > *Nmdar1*^{RNAi}	$W = 0.8522$	0.0391
	Kruskal–Wallis test	L_{dir}		$H = 15.27$	0.0005
	Dunn's multiple comparisons test	L_{dir}	T4 > *GFP* **vs**. T4 > *GluClα*^{RNAi}	$Z = 3.906$	0.0002
		L_{dir}	T4 > *GFP* **vs.** T4 > *Nmdar1*^{RNAi}	$Z = 1.318$	0.3748
**5f, ON**	Shapiro–Wilk test	Angular velocity	T4/T5 >	$W = 0.9418$	0.2839
	Shapiro–Wilk test	Angular velocity	*GluClα*^{RNAi}	$W = 0.9038$	0.0670
	Shapiro–Wilk test	Angular velocity	T4/T5 > *GluClα*^{RNAi}	$W = 0.9605$	0.5536
	Shapiro–Wilk test	Angular velocity	*Nmdar1*^{RNAi}	$W = 0.9478$	0.3915
	Shapiro–Wilk test	Angular velocity	T4/T5 > *Nmdar1*^{RNAi}	$W = 0.9701$	0.8000
	Brown–Forsythe test	Angular velocity		$F_{4, 88} = 1.589$	0.1843
	One-way ANOVA	Angular velocity		$F_{4, 88} = 7.715$	2.237×10^{-5}
	Holm–Šídák's multiple comparisons test	Angular velocity	T4/T5 > **vs.** T4/T5 > *GluClα*^{RNAi}	$t_{88} = 3.000$	0.0105
			GluClα^{RNAi} **vs.** T4/T5 > *GluClα*^{RNAi}	$t_{88} = 4.084$	0.0004
			T4/T5 > **vs.** T4/T5 > *Nmdar1*^{RNAi}	$t_{88} = 1.857$	0.1289
			Nmdar1^{RNAi} **vs.** T4/T5 > *Nmdar1*^{RNAi}	$t_{88} = 0.4669$	0.6417
**5f, OFF**	Shapiro–Wilk test	Angular velocity	T4/T5 >	$W = 0.9258$	0.0695
	Shapiro–Wilk test	Angular velocity	*GluClα*^{RNAi}	$W = 0.9532$	0.3398
	Shapiro–Wilk test	Angular velocity	T4/T5 > *GluClα*^{RNAi}	$W = 0.9039$	0.0488
	Shapiro–Wilk test	Angular velocity	*Nmdar1*^{RNAi}	$W = 0.9183$	0.0920
	Shapiro–Wilk test	Angular velocity	T4/T5 > *Nmdar1*^{RNAi}	$W = 0.9251$	0.1241
	Kruskal–Wallis test	Angular velocity		$H = 14.54$	0.0058
	Dunn's multiple comparisons test	Angular velocity	T4/T5 > **vs.** T4/T5 > *GluClα*^{RNAi}	$Z = 1.796$	0.2897
			GluClα^{RNAi} **vs.** T4/T5 > *GluClα*^{RNAi}	$Z = 3.488$	0.0019
			T4/T5 > **vs.** T4/T5 > *Nmdar1*^{RNAi}	$Z = 0.8056$	> 0.9999
			Nmdar1^{RNAi} **vs.** T4/T5 > *Nmdar1*^{RNAi}	$Z = 0.4493$	> 0.9999
**5i**	Shapiro–Wilk test	Fixation in front	T4/T5 >	$W = 0.9786$	0.9513
	Shapiro–Wilk test	Fixation in front	*GluClα*^{RNAi}	$W = 0.9274$	0.1751
	Shapiro–Wilk test	Fixation in front	T4/T5 > *GluClα*^{RNAi}	$W = 0.9447$	0.2696
	Shapiro–Wilk test	Fixation in front	*Nmdar1*^{RNAi}	$W = 0.9611$	0.7406
	Shapiro–Wilk test	Fixation in front	T4/T5 > *Nmdar1*^{RNAi}	$W = 0.9216$	0.4427
	Brown–Forsythe test	Fixation in front		$F_{4, 72} = 5.425$	0.0007
	Welch's ANOVA	Fixation in front		$W_{4.000, 27.14} = 12.78$	5.645×10^{-6}
	Dunnett's T3 multiple comparisons test	Fixation in front	T4/T5 > **vs.** T4/T5 > *GluClα*^{RNAi}	$t_{27.87} = 6.427$	2.337×10^{-6}
			GluClα^{RNAi} **vs.** T4/T5 > *GluClα*^{RNAi}	$t_{29.42} = 3.641$	0.0042
			T4/T5 > **vs.** T4/T5 > *Nmdar1*^{RNAi}	$t_{8.760} = 0.1015$	> 0.9999
			Nmdar1^{RNAi} **vs.** T4/T5 > *Nmdar1*^{RNAi}	$t_{15.65} = 0.6369$	0.9456

Supplementary Table 2. Statistical analyses of Extended Data Fig. 10.

Extended Data Figure	Statistical test	Measured variable	Experimental groups/comparisons	Test statistic	*P*
**10b**	Shapiro–Wilk test	Forward walking speed	T4/T5 >	$W = 0.9605$	0.6706
	Shapiro–Wilk test	Forward walking speed	*GluClα*^{RNAi}	$W = 0.9340$	0.2280
	Shapiro–Wilk test	Forward walking speed	T4/T5 > *GluClα*^{RNAi}	$W = 0.9422$	0.2403
	Shapiro–Wilk test	Forward walking speed	*Nmdar1*^{RNAi}	$W = 0.9454$	0.4913
	Shapiro–Wilk test	Forward walking speed	T4/T5 > *Nmdar1*^{RNAi}	$W = 0.8049$	0.0323
	Kruskal–Wallis test	Forward walking speed		$H = 4.563$	0.3352
**10d**	Shapiro–Wilk test	Forward walking speed	*R59E08-AD; R42F06-DBD*	$W = 0.8979$	0.1743
	Shapiro–Wilk test	Forward walking speed	*GluClα*^{RNAi}	$W = 0.9520$	0.5927
	Shapiro–Wilk test	Forward walking speed	*R59E08-AD; R42F06-DBD* > *GluClα*^{RNAi}	$W = 0.9309$	0.3139
	Brown–Forsythe test	Forward walking speed		$F_{2, 36} = 0.2397$	0.7881
	One-way ANOVA	Forward walking speed		$F_{2, 36} = 0.1688$	0.8453
**10f**	Shapiro–Wilk test	Fixation in front	*R59E08-AD; R42F06-DBD*	$W = 0.9553$	0.7126
	Shapiro–Wilk test	Fixation in front	*GluClα*^{RNAi}	$W = 0.9909$	0.9998
	Shapiro–Wilk test	Fixation in front	*R59E08-AD; R42F06-DBD* > *GluClα*^{RNAi}	$W = 0.9768$	0.9517
	Brown–Forsythe test	Fixation in front		$F_{2, 36} = 1.748$	0.1887
	One-way ANOVA	Fixation in front		$F_{2, 36} = 19.00$	2.327×10^{-6}
	Holm–Šídák's multiple comparisons test	Fixation in front	*R59E08-AD; R42F06-DBD* **vs.** *R59E08-AD; R42F06-DBD* > *GluClα*^{RNAi}	$t_{36} = 6.120$	9.599×10^{-7}
			GluClα^{RNAi} **vs.** *R59E08-AD; R42F06-DBD* > *GluClα*^{RNAi}	$t_{36} = 3.523$	0.0012
