# Supplementary material for: A biophysical account of multiplication by a single neuron
Source: Nature. 2022 Feb 23;603(7899):119–23. doi: 10.1038/s41586-022-04428-3 (PMC8891015; doi:10.1038/s41586-022-04428-3)
Supplement: Supplementary file 2 — Reporting Summary [file 41586_2022_4428_MOESM2_ESM.pdf]

nature portfolio

Corresponding author(s): Lukas N. Groschner, Alexander Borst

Last updated by author(s): Dec 15, 2021

# Reporting Summary

Nature Portfolio wishes to improve the reproducibility of the work that we publish. This form provides structure for consistency and transparency in reporting. For further information on Nature Portfolio policies, see our Editorial Policies and the Editorial Policy Checklist.

## Statistics

For all statistical analyses, confirm that the following items are present in the figure legend, table legend, main text, or Methods section.

| n/a | Confirmed | |
|---|---|---|
| ☐ | ☒ | The exact sample size ($n$) for each experimental group/condition, given as a discrete number and unit of measurement |
| ☐ | ☒ | A statement on whether measurements were taken from distinct samples or whether the same sample was measured repeatedly |
| ☐ | ☒ | The statistical test(s) used AND whether they are one- or two-sided<br>*Only common tests should be described solely by name; describe more complex techniques in the Methods section.* |
| ☐ | ☒ | A description of all covariates tested |
| ☐ | ☒ | A description of any assumptions or corrections, such as tests of normality and adjustment for multiple comparisons |
| ☐ | ☒ | A full description of the statistical parameters including central tendency (e.g. means) or other basic estimates (e.g. regression coefficient) AND variation (e.g. standard deviation) or associated estimates of uncertainty (e.g. confidence intervals) |
| ☐ | ☒ | For null hypothesis testing, the test statistic (e.g. $F$, $t$, $r$) with confidence intervals, effect sizes, degrees of freedom and $P$ value noted<br>*Give P values as exact values whenever suitable.* |
| ☐ | ☒ | For Bayesian analysis, information on the choice of priors and Markov chain Monte Carlo settings |
| ☒ | ☐ | For hierarchical and complex designs, identification of the appropriate level for tests and full reporting of outcomes |
| ☒ | ☐ | Estimates of effect sizes (e.g. Cohen's $d$, Pearson's $r$), indicating how they were calculated |

*Our web collection on statistics for biologists contains articles on many of the points above.*

## Software and code

Policy information about availability of computer code

| | |
|---|---|
| Data collection | Electrophysiological data were collected using pCLAMP 11 software (Molecular Devices). Behavioural data were collected with custom-written software in Python 2.7 (Python Software Foundation) as described by Bahl et al. (Nat. Neurosci., 2013). Micrographs were acquired using the Leica Application Suite X (Leica). |
| Data analysis | Data were analyzed with custom-written software in Python 3.7 (Python Software Foundation) using NumPy 1.15, Pandas 0.25, SciPy 1.3, Matplotlib 3.0, and pyABF 2.1 (https://pypi.org/project/pyabf/). Micrographs were processed in the Fiji distribution of ImageJ 2.0 (Schindelin et al., Nat. Methods, 2012). Simulation-based inference was performed in Python 3.7 using the software package sbi 0.8 (Tejero-Cantero et al., arXiv, 2020). Statistical tests were performed in Prism 9.2 (GraphPad). Custom-written code is available at https://dx.doi.org/10.17617/3.8g. |

For manuscripts utilizing custom algorithms or software that are central to the research but not yet described in published literature, software must be made available to editors and reviewers. We strongly encourage code deposition in a community repository (e.g. GitHub). See the Nature Portfolio guidelines for submitting code & software for further information.

## Data

Policy information about availability of data

All manuscripts must include a data availability statement. This statement should provide the following information, where applicable:

- Accession codes, unique identifiers, or web links for publicly available datasets
- A description of any restrictions on data availability
- For clinical datasets or third party data, please ensure that the statement adheres to our policy

Data is available through the Edmond Open Research Data Repository of the Max Planck Society at https://dx.doi.org/10.17617/3.8g.

# Field-specific reporting

Please select the one below that is the best fit for your research. If you are not sure, read the appropriate sections before making your selection.

☒ Life sciences ☐ Behavioural & social sciences ☐ Ecological, evolutionary & environmental sciences

For a reference copy of the document with all sections, see nature.com/documents/nr-reporting-summary-flat.pdf

# Life sciences study design

All studies must disclose on these points even when the disclosure is negative.

| | |
|---|---|
| Sample size | No sample size calculations were performed prior to experimentation. Sample sizes were chosen to match or exceed standard sample sizes in the field. For published patch clamp recordings from small neurons in the Drosophila visual system in vivo, sample sizes typically range from 2 to 17 cells (e.g. Gruntman et al., Nat. Neurosci., 2018; Behnia et al., Nature, 2014). Depending on cell type and experiment, this study used between 2 and 209 cells. Sample sizes in behavioural experiments (8 to 25 flies per genotype) were comparable to those of previous studies with similar experimental paradigms (e.g. Bahl et al., Nat. Neurosci., 2013; Strother et al., Neuron, 2017; Hindmarsh Sten et al., Nature, 2021). |
| Data exclusions | In patch clamp experiments, only cells with a measured resting potential more negative than –25 mV were characterized further. Two wild-type neurons were lost after the third glutamate application during patch clamp recordings for Fig. 2e and were excluded from the repeated-measures analysis. Six neurons were lost due to glutamate application during voltage clamp experiments shown in Fig. 2f and Extended Data Fig. 4b. The current-voltage relationships of those cells do not include all, but at least six, data points per cell. For behavioural experiments in Fig. 5e–i, the first 15 trials were used to equilibrate the temperature and to accustom the fly to the treadmill and were excluded from analyses. As inclusion criteria, we used a forward walking speed of ≥ 0.15 cm/s on a trial-by-trial basis and a minimum of ten trials per fly. For experiments in Extended Data Fig. 10d–f, each experiment consisted of 80 longer multi-stimulus trials, the first 10 of which were excluded. Here, only trials with a forward walking velocity of ≥ 0.40 cm/s and flies with at least 50 (20 for Extended Data Fig. 10d–f) of such trials were included in the analysis. To avoid possible turning bias, flies whose average turning deviated from zero by > 10 deg/s were excluded. |
| Replication | Data were generally consistent, as presented in the article. Behavioural experiments shown in Fig. 5h, i were replicated using a different, more specific split-GAL4 driver line and are presented in Extended Data Fig. 10e, f. |
| Randomization | For all experiments involving genetic perturbations, flies were grouped based on genotype. In open-loop behavioural experiments and all experiments involving two directions of visual stimuli, stimulus directions were alternated randomly; all remaining visual stimuli were presented in a strict sequence to allow for quick, intuitive interpretation. |
| Blinding | The investigators were not blind to genotype, because mating schemes involving conspicuous genetic markers and the occurrence of certain experimental genotypes at sub-Mendelian frequency made blinding impractical. Analyses were automated. |

# Reporting for specific materials, systems and methods

We require information from authors about some types of materials, experimental systems and methods used in many studies. Here, indicate whether each material, system or method listed is relevant to your study. If you are not sure if a list item applies to your research, read the appropriate section before selecting a response.

## Materials & experimental systems

| n/a | Involved in the study | |
|---|---|---|
| ☐ | ☒ | Antibodies |
| ☒ | ☐ | Eukaryotic cell lines |
| ☒ | ☐ | Palaeontology and archaeology |
| ☐ | ☒ | Animals and other organisms |
| ☒ | ☐ | Human research participants |
| ☒ | ☐ | Clinical data |
| ☒ | ☐ | Dual use research of concern |

## Methods

| n/a | Involved in the study | |
|---|---|---|
| ☒ | ☐ | ChIP-seq |
| ☒ | ☐ | Flow cytometry |
| ☒ | ☐ | MRI-based neuroimaging |

## Antibodies

| | |
|---|---|
| Antibodies used | Mouse anti-bruchpilot (nc82, Developmental Studies Hybridoma Bank) RRID: AB_2314866<br>Chicken anti-GFP antibody (Rockland) Cat. # 600-901-215S, RRID: AB_1537403<br>Atto 647N-conjugated goat anti-mouse IgG antibody (Rockland) Cat. # 610-156-040, RRID: AB_2614870<br>Alexa 488-conjugated goat anti-chicken IgY antibody (Invitrogen) Cat. # A-11039, RRID: AB_142924 |
| Validation | The mouse anti-bruchpilot antibody is widely used in the field of Drosophila neurobiology to stain presynaptic active zones. It was validated for use in Drosophila by Wagh et al. (Neuron, 2006).<br>The chicken anti-GFP antibody is a widely used antibody to label transgenically expressed GFP in the nervous system of the |

Drosophila (eg. Kim et al., Cell, 2017; Ribeiro et al., Cell, 2018). The manufacturer's data sheet documents the antibody's specificity for GFP using western blot and immunohistochemical analyses.

## Animals and other organisms

Policy information about studies involving animals; ARRIVE guidelines recommended for reporting animal research

| | |
|---|---|
| Laboratory animals | Drosophila melanogaster of the following genotypes were used:<br>P{R48A07-p65.AD}attP40, P{10XUAS-IVS-mCD8::GFP}su(Hw)attP5; P{VT046779-GAL4.DBD}attP2<br>P{R13E12-p65.AD}attP40/+; P{R59C10-GAL4.DBD}attP2/P{40XUAS-IVS-mCD8::GFP}attP2<br>P{R19F01-p65.AD}attP40/+; P{R71D01-GAL4.DBD}attP2/P{40XUAS-IVS-mCD8::GFP}attP2<br>P{R48A07-p65.AD}attP40, P{10XUAS-IVS-mCD8::GFP}su(Hw)attP5; P{R13F11-GAL4.DBD}attP2<br>P{R26H02-p65.AD}attP40/+; P{R29G11-GAL4.DBD}attP2/ P{40XUAS-IVS-mCD8::GFP}attP2<br>P{R42F06-p65.AD}attP40, P{10XUAS-IVS-mCD8::GFP}su(Hw)attP5; P{VT037588-GAL4.DBD}attP2<br>P{TRiP.HMC03585}attP40/P{R42F06-p65.AD}attP40, P{10XUAS-IVS-mCD8::GFP}su(Hw)attP5; P{VT037588-GAL4.DBD}attP2/+<br>P{TRiP.HMS02199}attP2/P{R42F06-p65.AD}attP40, P{10XUAS-IVS-mCD8::GFP}su(Hw)attP5; P{VT037588-GAL4.DBD}attP2/+<br>P{UAS-Dcr-2.D}2/P{TRiP.HMC03585}attP40 ; P{GMR39H12-GAL4}attP2/+<br>P{UAS-Dcr-2.D}2/+; P{GMR39H12-GAL4}attP2/P{TRiP.HMS02199}attP2<br>P{R59E08-p65.AD}attP40/P{TRiP.HMC03585}attP40; P{R42F06-GAL4.DBD}attP2/+<br>P{R59E08-p65.AD}attP40/+; P{R42F06-GAL4.DBD}attP2/P{TRiP.HMS02199}attP2<br>P{UAS-Dcr-2.D}2/P{10XUAS-IVS-mCD8::GFP}su(Hw)attP5; P{GMR39H12-GAL4}attP2/+<br>P{R59E08-p65.AD}attP40/P{10XUAS-IVS-mCD8::GFP}su(Hw)attP5; P{R42F06-GAL4.DBD}attP2/+<br>All experiments were carried out on female flies bearing at least one wild-type allele of the white gene. For electrophysiological experiments, animals were aged 2–24 hours post-eclosion; for behavioural experiments, animals were aged 1–5 days. |
| Wild animals | No wild animals were used in this study. |
| Field-collected samples | No field-collected samples were used in this study. |
| Ethics oversight | No ethical approval was required for research on Drosophila melanogaster. |

Note that full information on the approval of the study protocol must also be provided in the manuscript.
